# Transforming Waste into Wealth: Advanced Carbon-Based Electrodes Derived from Refinery and Coal By-Products for Next-Generation Energy Storage

**DOI:** 10.3390/molecules29092081

**Published:** 2024-04-30

**Authors:** Ar Rafi Ferdous, Syed Shaheen Shah, Syed Niaz Ali Shah, Bashir Ahmed Johan, Md Abdullah Al Bari, Md. Abdul Aziz

**Affiliations:** 1 Department of Petroleum and Mining Engineering, Chittagong University of Engineering and Technology, Chittagong 4349, Bangladesh; arrafiferdous@gmail.com; 2 Department of Material Chemistry, Graduate School of Engineering, Kyoto University, Nishikyo-ku, Kyoto 615-8520, Japan; 3 Innovation and Technology Transfer, King Fahd University of Petroleum and Minerals, Dhahran 31261, Saudi Arabia; niazalianalyst@gmail.com; 4 Materials Science and Engineering Department, King Fahd University of Petroleum & Minerals, Dhahran 31261, Saudi Arabia; johan105105@gmail.com; 5 Department of Mechanical Engineering, King Fahd University of Petroleum and Minerals, Dhahran 31261, Saudi Arabia; mdabdullahal.bari@kfupm.edu.sa; 6 Interdisciplinary Research Center for Hydrogen Technologies and Carbon Management (IRC-HTCM), King Fahd University of Petroleum & Minerals, KFUPM Box 5040, Dhahran 31261, Saudi Arabia

**Keywords:** carbon-based electrodes, waste-to-energy conversion, electrochemical energy storage, sustainable material development, activated carbon precursors

## Abstract

This comprehensive review addresses the need for sustainable and efficient energy storage technologies against escalating global energy demand and environmental concerns. It explores the innovative utilization of waste materials from oil refineries and coal processing industries as precursors for carbon-based electrodes in next-generation energy storage systems, including batteries and supercapacitors. These waste-derived carbon materials, such as semi-coke, coal gasification fine ash, coal tar pitch, petroleum coke, and petroleum vacuum residue, offer a promising alternative to conventional electrode materials. They present an optimal balance of high carbon content and enhanced electrochemical properties while promoting environmental sustainability through effectively repurposing waste materials from coal and hydrocarbon industries. This review systematically examines recent advancements in fabricating and applying waste-derived carbon-based electrodes. It delves into the methodologies for converting industrial by-products into high-quality carbon electrodes, with a particular emphasis on carbonization and activation processes tailored to enhance the electrochemical performance of the derived materials. Key findings indicate that while higher carbonization temperatures may impede the development of a porous structure, using KOH as an activating agent has proven effective in developing mesoporous structures conducive to ion transport and storage. Moreover, incorporating heteroatom doping (with elements such as sulfur, potassium, and nitrogen) has shown promise in enhancing surface interactions and facilitating the diffusion process through increased availability of active sites, thereby demonstrating the potential for improved storage capabilities. The electrochemical performance of these waste-derived carbon materials is evaluated across various configurations and electrolytes. Challenges and future directions are identified, highlighting the need for a deeper understanding of the microstructural characteristics that influence electrochemical performance and advocating for interdisciplinary research to achieve precise control over material properties. This review contributes to advancing electrode material technology and promotes environmental sustainability by repurposing industrial waste into valuable resources for energy storage. It underscores the potential of waste-derived carbon materials in sustainably meeting global energy storage demands.

## 1. Introduction

In the quest to meet the rising global energy demands amidst depleting fossil fuel reserves, developing renewable energy sources and enhancing energy storage technologies have become increasingly critical [1]. This urgency is underscored by the pivotal role of energy storage devices, such as batteries and supercapacitors, which offer efficient, reliable, and sustainable energy storage solutions. The efficacy of these devices is fundamentally influenced by their electrode materials, which dictate their performance, durability, and environmental impact. Therefore, searching for electrode materials that balance efficiency, superior electrochemical properties, and sustainability is at the forefront of modern research endeavors [2,3].

Electrode materials are crucial for determining the energy storage capacity, power density, charge–discharge rates, and stability of batteries and supercapacitors. Traditional electrode materials, predominantly metals and metal oxides, face challenges regarding cost, availability, and environmental implications [4]. These challenges have led to a shift towards carbon-based materials, noted for their versatility, high surface area, and exceptional conductivity. However, the sustainability of carbon sources is a pressing issue, directing attention to waste materials from oil refineries and coal processing plants as sustainable resources [5]. This innovative direction leverages waste-derived carbon materials, such as semi-coke, coal gasification fine ash, coal tar pitch, and petroleum coke (pet coke) [3,6,7]. Traditionally seen as environmental liabilities, these by-products are now valued for their potential in green energy solutions. These materials are transformed into high-quality carbon-based electrodes through processes like carbonization and activation, addressing waste management issues while meeting the demands for efficient energy storage [8,9].

Carbon-based materials are closely associated with a friendly environment and are critical for environmental remediation. Using carbon-based materials is sustainable and environmentally friendly, characterizing their application in almost every aspect of environmental science, renewable energy, and pollution prevention strategies [10,11,12,13,14]. For instance, carbon-based structures are beneficial for the generation and storage of renewable energy, as well as for purification and remediation in environmental science. In addition, materials such as biochar based on biomass are affordable, environmentally friendly, and abundant and can be used in environmental remediation. Carbon-based materials based on activated carbon are environmentally friendly and have been determined to be a potential base material for a supercapacitor [15,16]. Carbon-based nanomaterials, such as sorbents, high-flux membranes, environmental sensors, and pollution prevention strategies, have massive optimization for ecological applications [12,17]. Developing porous carbons from various biomass offers promise for economic and environmentally friendly materials. Also, carbon-based materials promote environmental sustainability through the remediation of paddy soils [18]. Therefore, carbon-based materials address environmental challenges and provide solutions for more sustainable measures.

The efficiency of the energy storage application is closely associated with the electrode material’s pore size and surface area. Based on prior studies, controlling pore size is critical in determining the efficiency of energy storage applications, specifically supercapacitors and capacitive deionization. Recent studies discovered that the complete or step-by-step boosting of a supercapacitor’s capacitance at higher pressures could be elucidated by pore-filling efficiency, which should rely on the ion size in the electrolyte solution and the gap of the electrode pore [19]. Furthermore, it was stated that the pore size requirement varied based on the dimensions of the electrolyte instead of on the optimal pore size of the electrode material to improve electrochemical supercapacitors [20]. Additionally, it is crucial to note that outstanding capacitive deionization electrode materials must have a vast ion-accessible space for a specific surface and a superior pore size distribution for optimal execution [21]. Ironically, the ion dimension and pore size were identified as factors to consider when discussing the capacitance behavior of electrode materials. It was confirmed by earlier studies that exceedingly close pore sizes will lead to saturation on the ground, while elevated pore dimensions will reduce the utilization of electrode materials [22]. Furthermore, the suggested carbon electrode materials should have a substantial specific area, an evolving pore structure, and outstanding interpolar action to improve the absorption, intuition, and transfer of the electrolyte ion [23]. Therefore, it can be deduced that the electrode material’s pore size and surface area play a critical role in the efficiency of energy storage applications.

The transition from traditional to innovative electrode materials also brings into focus the specifics of battery chemistry and its environmental impact. For instance, lithium-ion batteries (LIBs) utilizing lithium hexafluorophosphate (LiPF_6_) as an electrolyte and materials like lithium cobalt oxide (LiCoO_2_) for the cathode offer high energy density but raise concerns over resource scarcity and recycling challenges [24,25,26]. Similarly, despite being among the “greenest” battery systems due to their well-established recycling processes, lead–acid batteries achieve only about 25% of the theoretical energy density. This limitation is attributed to the presence of heavy materials in electrolytes (such as sulfuric acid) and electrodes (like lead) and the necessity of several inactive components (e.g., separator and container) for a complete system. However, their eco-friendliness and cost-effectiveness cannot be overlooked [27,28].

The advancement in waste-derived carbon materials for electrodes represents a significant stride towards overcoming these challenges. Research demonstrates that by manipulating factors such as carbonization temperature, activating agents, and heteroatom doping, the electrochemical properties of these carbon materials can be finely tuned [10,29,30]. Despite these promising developments, challenges persist in fully exploiting the potential of waste-derived carbon materials for energy storage, necessitating a deep understanding of their microstructural characteristics and electrochemical performance [31]. Exploring waste-derived carbon materials for electrode development offers a solution to environmental and waste management issues and enhances the performance of batteries and supercapacitors [32,33]. This approach marks a significant step towards a sustainable energy future by aligning the goals of renewable energy adoption with environmental stewardship.

## 2. Carbon Materials for Electrochemical Energy Storage Technologies

The evolving landscape of electrochemical energy storage technologies, including batteries, supercapacitors, and hydrogen evolution reaction (HER) systems, critically hinges on the innovative development of electrode and electrocatalyst materials. The efficacy of these materials in storing and converting electrical energy into chemical energy is fundamentally linked to their chemical compositions and structural integrity. This section delves into the paradigm of utilizing waste-derived carbon materials for electrode fabrication, highlighting their transformative potential in advancing next-generation energy storage solutions [34,35,36].

### 2.1. Electrochemical Storage Systems and Carbon-Based Nanostructures

Electrochemical storage systems are pivotal in the modern energy landscape, underpinning the functionality of batteries, supercapacitors, and HER devices. The efficiency of these systems in storing and converting energy is greatly influenced by the electrochemical properties of their constituent materials. Carbon-based nanostructures have emerged as frontrunners in energy storage and conversion applications with their vast surface area, tunable morphologies, and robust thermal, chemical, and physical properties. Graphene, carbon nanotubes (CNTs), and fullerenes, all exhibiting sp^2^ hybridization, are extensively studied for their superior electrochemical characteristics [37,38,39].

Surface functional groups on carbon-based electrode materials derived from refinery and coal by-products are crucial in shaping their electrochemical performance. These groups, often oxygen-, nitrogen-, or sulfur-based, affect capacitance, electrical conductivity, and durability in energy storage applications [40]. Oxygen-containing groups, like hydroxyl, carboxyl, and carbonyl, boost hydrophilicity, improving interaction with electrolytes and ion transport. Nitrogen-containing groups, such as pyridinic and pyrrolic nitrogen, enhance electron-donating capability, improving conductivity. Sulfur-containing groups can increase redox activity, aiding energy storage efficiency [29]. The synthesis process determines the type and distribution of these functional groups. High-temperature treatments can reduce oxygen-based groups, affecting hydrophilicity, while chemical activation methods allow for precise control over functional group addition. Post-synthesis surface modifications can further refine the electrochemical characteristics. Functional groups significantly impact various energy storage applications. In supercapacitors, oxygen and nitrogen groups contribute to pseudocapacitance, increasing energy storage capacity and enabling rapid charge/discharge cycles [40]. Understanding and manipulating these functional groups in carbon-based electrodes derived from refinery and coal by-products is critical in advancing next-generation energy storage. By controlling the synthesis and post-synthesis processes, researchers can optimize these materials for improved efficiency, stability, and energy storage capacity [40].

### 2.2. Supercapacitor Electrode Materials

Carbon materials are considered efficient supercapacitor electrode materials due to their high surface area and porous structure, facilitating rapid ion transport and enhancing charge storage capacity. Additionally, biomass resources’ inherent sustainability and abundance make these carbon materials cost-effective and environmentally friendly for energy storage applications [15,16,41]. The electrode material plays a quintessential role in dictating the performance metrics of supercapacitors, including storage capacity, operating voltage, and energy density. The electrode’s accessible surface area primarily determines the charge storage capability of a supercapacitor. Energy storage occurs through the distribution of ions near the electrode surfaces, forming an electric double layer (EDL). The electrostatic attraction between the two electrodes characterizes the electric double-layer capacitor (EDLC) behavior, whereas pseudocapacitors (PCs) leverage quick and reversible faradaic reactions on the electrode surface for charge storage [42]. Due to the electrostatic charge transfer processes, PCs, utilizing a Faradaic charge storage mechanism, demonstrate higher energy densities than EDLCs [43,44].

### 2.3. Batteries and Porous Carbon Materials

In batteries, the charging and discharging mechanism is intricately governed by the intercalation and deintercalation of lithium ions within the electrode materials. With its ample lattice spacing, graphite is an ideal cathodic material, facilitating the efficient travel of lithium ions through the electrolyte toward the cathode. The porosity of the electrode material significantly impacts the battery’s performance, as a larger surface area provides more active sites for lithium–ion adsorption. Hence, highly porous carbon materials from waste sources offer an enhanced interface for lithium–ion interaction, boosting the charging and discharging efficiency [45].

Biomass-derived carbon materials have gained significant attention in electrochemical energy storage due to their structural diversities, adjustable physical/chemical properties, and environmental friendliness. These materials, including porous carbon and nanostructured carbons, exhibit excellent conductivity and rich porosity, making them promising for energy storage, catalysis, and environmental applications [6,46]. The typical self-doped heteroatoms in biomass-derived carbons, such as N, O, and S, can be modified by single doping or co-doping, enhancing their performance as electrode materials [47]. Furthermore, recent studies have shown that hybrids of biomass-derived carbon and metallic compounds, such as transition metal oxides, nitrides, and sulfides, have demonstrated superior performance as anode materials for sodium-ion batteries [48]. Biomass-derived carbons have been investigated as hosts for sulfur in the context of sulfur-based aqueous batteries. For instance, 3D hybrid materials, such as chemically coupled nickel sulfide and hollow carbon spheres, have shown unique advantages as sulfur hosts, with high polysulfide adsorption capacity and good stability during battery operation. Additionally, heteroatom-doped carbon materials have been widely utilized as sulfur hosts to improve the electrochemical performance of lithium–sulfur batteries. Moreover, non-polar carbon-based materials have been explored to address sulfur hosts in room-temperature sodium–sulfur batteries [49,50].

### 2.4. Advancing Carbon Electrodes from Waste Sources

Leveraging waste materials from oil refineries and coal processing as precursors for carbon-based electrodes embodies a strategic shift towards sustainable and efficient energy storage technologies. These waste-derived carbon materials, processed through carbonization and activation, exhibit tailored electrochemical properties crucial for high-performance energy storage applications [51]. The optimization of these processes, alongside the strategic incorporation of heteroatoms, can significantly enhance the electrochemical activity, conductivity, and structural stability of the resulting electrodes. This mitigates waste disposal challenges and positions waste-derived carbon materials as viable alternatives to traditional electrode materials, marking a pivotal step in the quest for green and efficient energy storage solutions [52,53,54].

Exploring waste-derived carbon materials for electrode applications presents a promising avenue for addressing the dual challenges of environmental sustainability and energy storage efficiency. By harnessing the intrinsic properties of carbon-based nanostructures and optimizing their electrochemical performance, we stand on the brink of a new era in energy storage technologies. This paradigm shift not only exemplifies the transformative potential of waste materials but also underscores the critical role of innovative material science in powering the future of energy storage.

## 3. Refinery Wastes and Coal as a Source of Electrode Materials

**Figure 1:**
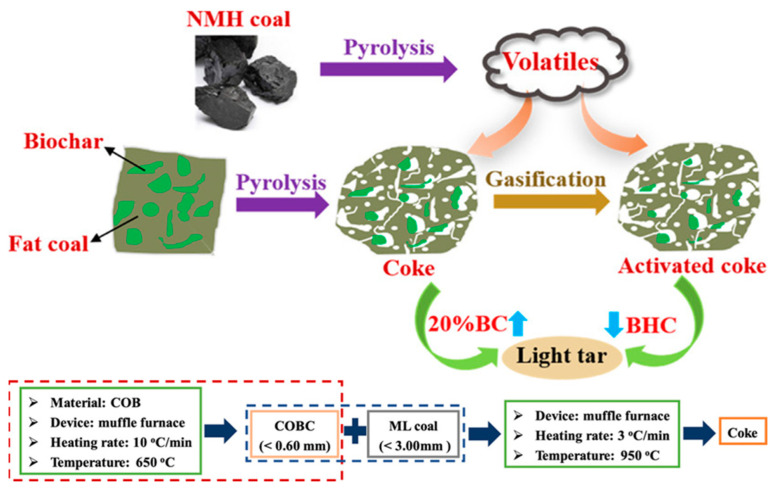
Schematic representation of coal pyrolysis and gasification processes with subsequent activation of the resulting coke to create catalytically active porous carbon materials derived from biochar–coal blends, used for upgrading coal pyrolysis volatiles. Reproduced with permission [58]. Reproduced under the term CC-BY-NC-ND. Copyright 2021, Wang et al., American Chemical Society.

Scientists have been desperately searching for appropriate precursors to synthesize carbon with excellent electrochemical properties. Many of them include biomass for electrode preparation for supercapacitors and batteries. Although they can be obtained at a lower cost and are readily available, the main drawback of these materials is the low carbon yield compared to the counterparts obtained from refineries and mining industries. Biomass materials generally have a carbon content of around 50–55%, for example, almond shell (49.94%), bamboo (47.65%), coconut shell (52.37%), corn-cob (48.22%), oak wood (50.13%), rice hull (38.86%), and walnut shell (49.95%) [55]. On the other hand, the carbon content in petroleum residue such as coke (>90%) [3], bituminous coal (83.21%) [56], and marine heavy fuel oil (86%) [57] is much higher and can produce high yield carbon materials for electrochemical applications. Naomaohu (NHM) long flame coal with high volatiles contains 74.35% carbon, and Malan fat coal, rich in metaplast, carries ~86% carbon [58]. Figure 1 illustrates a process diagram for converting different types of coal (NHM coal and Fat coal) into more valuable products through pyrolysis and gasification. In the process, NMH coal undergoes pyrolysis to produce volatiles, while fat coal is subjected to pyrolysis and gasification. The pyrolysis of both coals yields coke, which can be further activated to produce activated coke. This activated coke is then used with light tar to create biochar–coal blends at different ratios for catalytic purposes. Below the central diagram, additional details are provided for the experimental conditions, such as the type of device (muffle furnace), particle size of coal, heating rate, and temperature for both the creation of coke from fat coal and the conversion to activated coke. Various carbon content of biomass precursor materials depends on their origin and subsequent growth in a particular climate and soil. Therefore, the carbon content can vary widely, and supply can be interrupted. On the other hand, materials such as coal, coke, tar, pitch, and heavy oil residue are mostly considered waste or low-value materials [59]. They also have a significant environmental footprint as they are being unconsciously released and washed in the water bodies [60].

Oil refineries provide almost all fuels through various refinery processes that we use daily. Apart from all the valued products, some low-valued heavy by-products and wastes are also generated, such as pet coke and asphaltenes, which can be utilized as raw materials to prepare various energy storage devices [61]. In addition, coal industry-produced by-products such as coal tar, coal pitch, and coal liquefication residue have also attracted the attention of scientists in recent years due to their abundance and low cost [62]. The main advantage lies in their structure, which supports easy fabrication of the final carbonaceous product through polymerization as they contain various polycyclic aromatic hydrocarbons [63,64]. Although biomass precursors are widely used in a good number of research due to their low environmental footprint, availability, and low cost, they exhibit some issues. The structural manipulation of the final carbon product derived from these biomass precursors is quite tricky because the significant attributes of carbonaceous products mainly depend on the raw biomass, which differs according to their place of origin and season [65]. 

Asphaltenes are classified as complex organic matter, including sulfur, nitrogen, oxygen, and carbon. This part of the crude oil is nonpolar and nonvolatile. They are soluble in aromatics (benzene and toluene) but insoluble in alkanes (normal pentane and normal heptane). Certain crude oils and residuals have a black hue due to asphaltenes, which do not precipitate or flocculate [66]. Pet coke is also called green coke in its unprocessed state and is frequently created as a by-product of refining crude oil, particularly from heavy crudes. It is a by-product produced during cracking in its solid state [67,68]. As a waste product of the industry, it is frequently heaped high in storage. However, the stored pet coke is burned to produce heat or power in many countries. Since it is more carbon-intensive than burning coal, it produces carbon-based toxic gases, which have become a major environmental issue. It has recently been used as a carbon source for various metallurgical uses, such as producing anodes for graphite electrodes, making it a vital industrial by-product connecting the oil and metallurgical sectors [69].

The complex hydrocarbon combination known as coal tar is created when coal is pyrolyzed. It is the by-product produced during the destructive distillation to produce coal gas or coke from coal. It is a black, smoky, or naphthenic semisolid or viscous liquid. The initial coal content and the pyrolytic distillation method will affect coal tar’s composition [70]. Further distillation of coal tar produces coal tar pitch, which is semi-solid or solid, thick and black, and contains primarily aromatic hydrocarbons. The final product of the coal carbonization process is coal coke, often called metallurgical coke. It contains carbon impurities as most of the volatile part of coal is removed during the carbonization process [71]. Coal tar products treat conditions like psoriasis in skin care products. Coal tar, coal tar pitch, and volatiles from car tar pitch are used or generated in several sectors, including coking, rubber manufacturing, roofing, aluminum smelting, and road paving [72]. Coke is widely utilized in blast furnaces as fuel to produce steel from iron ore (iron oxide). 

The leftovers from refining crude oil are heavy fuel oils (HFOs). This mainly comprised the nondistillable residue from the atmospheric distillation process, such as the portion of crude oil that did not boil over 1050 °F to 1100 °F. Although it was the situation before the 1970s and now, it has changed to a large extent. The majority of refiners now also use vacuum distillation methods along with more sophisticated procedures to extract more value from residues (such as thermal and catalytic cracking, visbreaking, and coking), which allow them to “squeeze” even lighter and more desired products out of the residual material from atmospheric distillation. Therefore, increased sulfur, ash (often linked to catalyst particles), and metals are found in HFOs with increased viscosity, pour point, and water content. Due to its low cost, it is primarily utilized as a fuel for marine propulsion systems, power plants, and desalination plants, and it is gaining interest despite its grave environmental repercussions [73]. It is often referred to as marine diesel due to its wide variety of applications in marine propulsion systems [74]. The HFO combustion process in these sectors produces a significant amount of ash (heavy fuel oil ash) that contains metallic oxide. It has over twenty-five distinct metals in it, mostly Ni, Fe, Ca, Na, Al, and V, according to reports, along with impurities. This waste material contains heavy metals, organic (aliphatic and aromatic hydrocarbons), and inorganic (S and N compounds) pollutants; therefore, improper treatment during traditional land disposal might have long-term adverse effects on the ecosystem [73].

All these materials have been successfully used in preparing carbonaceous materials (AC, CNTs, carbon sheets, etc.) for various applications in relation to toxin adsorption, pollutant removal, etc. [73,75,76]. Similarly, they can be suitable for producing electrodes for energy storage devices. Some recent research has proved that these low-value materials can be precursors for these carbon materials, which can be used as electrodes in batteries and supercapacitors in various energy storage devices. Table 1 summarizes recent studies on preparing carbon-based electrodes using materials collected from multiple refineries and coal treatment plants. Their performance as electrode material for supercapacitor applications has also been outlined in Table 2.

### 3.1. Preparation of Carbon Materials 

It is possible to effectively construct flexible N, S co-doped cross-linked carbon nanofibers from Preasphaltene (PA) by regulating the stabilization temperature and timings and then carbonizing the fibers using (NH_4_)_2_SO_4_. PA obtained from coal liquefication residue (CLR) is an efficient and cheap carbon precursor with a high carbon content. The resultant N, S co-doped cross-linked carbon nanofibers sample (N, S-CLPACF) has high electrical conductivity, a significant SSA, and excellent N and S heteroatoms concentrations. N, S-CLPACF has pore volumes of 0.280 cm^3^/g and SSA of 589 m^2^/g, which are much larger than those of PACF and CLPACF [77]. The ashes found in CLR are once more utilized to create porous carbon. The porosity of the residue was improved by potassium hydroxide (KOH) during the carbonization process, as demonstrated by the porous structures of HPC-600, HPC-700, and HPC-800 (180.6 m^2^/g) in comparison to CLR-700 (121.6 m^2^/g). When CLR-700 was provided after the CLR had carbonized, uneven and lumpy particles with no visible porosity structure were noticed [78]. 

Despite being one of the most sophisticated hybrid energy storage devices, the imbalance of dynamics and capacity between the anode and cathode electrodes impedes the further development of lithium-ion capacitors (LICs). g-C_3_N_4_ was used to manufacture anthracite-based nitrogen-doped porous carbon materials, and their effectiveness was assessed [79]. Adjusting the amount of g-C_3_N_4_ allows its porosity architectures and heteroatom doping to be successfully controlled. CTK-1.0 had a maximum pore volume of 0.77 cm^3^/g and a maximum surface area of 1673.5 m^2^/g. The porous and linked carbon nanosheets allowed for a significant specific surface area to be in contact with the electrolyte. At the same time, the nitrogen-doping structure provided additional active sites for Li^+^ adsorption and pseudocapacitance [80]. A two-step heat treatment process created oxygen self-doped hierarchical porous carbons (OHPCs) utilizing KOH as the activation agent and CLR as the raw material. When OHPC was used as the electrode material for a supercapacitor, the oxygen-containing functional group experienced a redox reaction throughout the energy storage process to raise the pseudocapacitance. The doping oxygen species in carbon provide a quick electron transfer channel and improved adsorption to the electrolyte. The specific surface area of OHPCs rose with increasing activation temperature, going from 2007 m^2^/g of OHPC-600 to 2691 m^2^/g of OHPC-700, ultimately reaching 3102 m^2^/g of OHPC-800 [81].

Porous carbon materials were created by thermally treating coal-based green needle coke to 750 °C, 850 °C, or 950 °C [82]. The raw needle-coke had a BET surface area of just 3.0 m^2^/g; however, upon activation, notably at 750 °C (807.69 m^2^/g), this surface area significantly increased. Green needle coke’s amorphous carbon might be readily activated even at very low temperatures (750 °C) because of the carbon matrix’s faulty sites, which caused a highly porous structure to develop. The specific surface area would drop, and the average pore diameter would rise as the treatment temperature increased to 850 °C, which would cause the pore walls to collapse to generate larger pores [83]. Using ultrasonic assistance, subbituminous coal was processed chemically to create activated carbon (AC) with a high specific surface area and porosity [84]. The outcome unequivocally showed that the structural characteristics of AC are impacted by chemical activation aided by ultrasonic technology. Under ultrasonic irradiation, it was found that vibration and cavitation activity increased the porosity and energy of the coal surface. The development of mesopores coincided with the pore volume growth. The AC’s surface area and pore volume were both enhanced by ultrasonic vibration [85]. The augmentation of the surface area to 789.910 m^2^/g with a total pore volume of 0.103 cm^3^/g for JCRA2 explained the impacts of the ultrasonic-assisted chemical activation of these coals [84].

**Figure 2:**
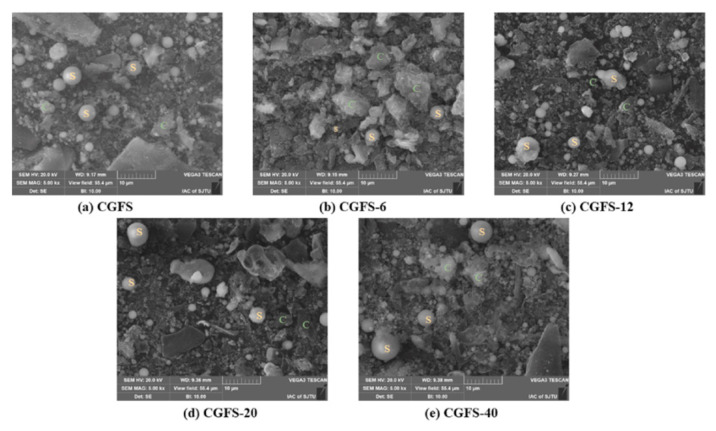
SEM micrographs for the CGFS and activated CGFS samples. C indicates unburned carbon; S denotes spheres of inorganic minerals. Reproduced with permission [87]. Copyright 2023, Elsevier.

In a study, coal-based carbon dots produced AC using an efficient combination of oxidation and KOH activation at a low KOH level. In producing coal-based AC, the one-step activation process frequently faces challenges when adding surface-functional groups and requires a high KOH dose. Thus, this approach was used. With a large specific surface area (1207 m^2^/g), pore volume (1.346 m^3^/g), and an abundance of surface-functional groups, the resulting AC might offer a large number of active sites and excellent wettability, leading to increased specific capacitance and improved electrochemical performances [86]. Scientists have used coal gasification fine slag (CGFS) to create AC because of its greater output of residual carbon and possible leak danger. Using low-temperature air activation, an AC material was made from CGFS, which has an abundant carbon content (about 33.61 wt.%). The particles exhibited spherical, blocky, fluffy particles and porous shapes, as shown in Figure 2. The CGFS-20 samples showed a 21.5% improvement in specific surface area over the CGFS samples, with a maximum value of 300.84 m^2^/g. This suggests that appropriate activation can effectively increase the samples’ specific surface area and enhance their adsorption capacity. Moreover, the overall pore volume increased by 7.8% (0.3093 m^3^/g) [87].

Recently, researchers revealed the combined use of carbon compounds based on hydrocarbons and biomass. The carbon precursor for the synthesis of 3D hierarchical porous carbons (HPCs) was the soluble sections from the co-thermal dissolution (CTD) of coal and rice husk (RH). One crucial element influencing the properties of carbon-based materials is the makeup of carbon precursors. It was discovered that organic materials, including N in carbon precursors, could increase conductivity, and compounds containing O could increase pseudocapacitance. Research on the connections between carbon precursors’ molecular makeup and electrochemical properties might help choose and optimize carbon precursors, enhancing carbon materials’ electrochemical performance. In this case, the specific surface area of carbon materials decreased due to the collapse of pores caused by a high carbonization temperature. KOH and ZnO broadly alter the morphology and pore structure [88].

Two significant challenges for lithium-ion capacitors are the mismatches in electrochemical kinetics and capacity between the anode and cathode. A study strengthened AC and graphitized carbon as cathode and anode by applying graphene coating and electrochemical pre-lithiation [89]. Anthracite was used as a raw material to manufacture graphitized carbon with porosity and AC with a particular surface area. Both of these products are subsequently coated using graphene obtained from anthracite. The graphitic carbon had the most significant total pore volume of 0.07 cm^3^/g and a maximum specific surface area of 18.7 m^2^/g. In contrast, the AC had a maximum specific surface area of 2256 m^2^/g and a pore volume of 1.21 cm^3^/g [90]. Coal coke was processed in two steps to create capacitive carbon. After pre-treating coal coke with a solution of hydrochloric and nitric acids, potassium hydroxide was used to activate the material at a high temperature chemically. With a large specific surface area of 1633 m^2^/g and abundant oxygen and nitrogen dopants, the prepared coal-carbon was advantageous for Faradic and electric double-layer processes [91]. 

**Figure 3:**
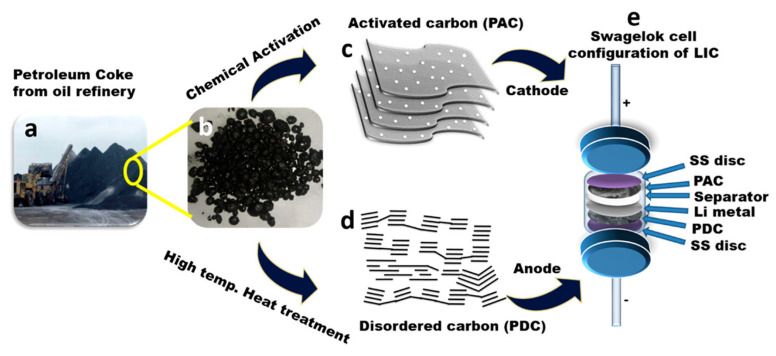
Process of fabricating lithium-ion capacitors from petroleum coke waste. (**a**) Petroleum coke waste sourced from oil refineries. (**b**) Close-up view of petroleum coke granules. (**c**) Diagram showing the production of activated carbon from petroleum coke through KOH chemical activation. (**d**) Diagram illustrating the creation of disordered carbon from petroleum coke via high-temperature heat treatment. (**e**) Diagram depicting a lithium-ion capacitor constructed with carbon materials derived from petroleum coke, housed within a Swagelok-type cell configuration. Reproduced with permission [92]. Copyright 2021, American Chemical Society.

High-energy and high-power LICs were considered appealing for advanced energy storage applications. A disordered carbon anode with a low surface area and an AC cathode with a high surface area were synthesized using pet coke, an inevitable high-carbon industrial by-product, as a single carbon source. The activated sample was determined to have a BET surface area, pore size, and pore volume of 1952 m^2^/g, 2.2 nm, and 1.1 cm^3^/g, respectively. With a BET surface area of 1.5 m^2^/g, disordered carbon suggests the presence of mesopores and little to no micropores. While disordered carbon lacked this sheet topology, AC generated from pet coke exhibits a sheetlike form [92]. Figure 3a–e depicts transforming pet coke, a waste by-product from oil refineries, into a component for LICs. Starting with the raw petroleum coke, the material undergoes a chemical activation process with KOH to produce AC. This AC serves as the cathode material in the LIC. Petroleum coke is subjected to high-temperature heat treatment to produce disordered carbon, used as the anode material. Finally, the LIC is assembled in a Swagelok cell configuration, with the pet coke-derived AC (PAC) at the cathode, the disordered carbon (PDC) at the anode, and a separator between them. Lithium metal is incorporated, and stainless steel (SS) disks complete the assembly, which ensures good electrical contact and structural integrity.

Scientists were desperately searching for more effective ways to employ high-sulfur pet coke in synthesizing carbon materials for supercapacitor applications, as it has extremely limited uses other than fuel for burning. Without external sulfur, a sulfur-doped HPC was made directly from high-sulfur pet coke utilizing a one-step procedure. MnO_2_ nanowires were added to HPC to create a porous composite material that was then used to develop high-performance supercapacitors. After MnO_2_ doping, HPC’s BET surface area and total pore volume dropped to 2079 m^2^/g and 1.04 cm^3^/g, respectively, from 3318 m^2^/g and 1.67 cm^3^/g, respectively [93].

Multilayer highly curved fullerene shells make up carbon nano-onions (CNOs), which have interesting electrochemical properties when used as supercapacitor electrode materials. Moreover, the many nanovoids in inner space and the sp^2^-hybridized carbon shell of CNOs facilitate the loading of pseudocapacitive species. Pitch-coated pet coke might be easily annealed to produce CNOs on a gram-scale for use in supercapacitors. With a consistent particle size of around 5–30 nm, an exceptional surface area of up to 2665.5 m^2^/g, and a sizable pore volume of 1.2 cm^3^/g, CNOs were advantageous for electronic and ionic transport in supercapacitors. Their morphology resembled a sponge, with macropores ranging in diameter from 100 to 700 nm [94]. 

With its valuable applications in energy devices, producing multi-heteroatom self-doped graphene structures from waste materials will be an effective solution to satisfy energy requirements. It is proposed that graphene can be an alternative to AC for energy storage devices. Debabrata Mandal and colleagues suggested a unique, high-yield synthetic method to create a multi-heteroatom self-doped extremely porous graphene nanosheet from pet coke [95]. Using co-activation as a synthesis method, nickel sulfide/hierarchical porous carbon (Ni_3_S_2_/HPCs) was effectively synthesized from the Ni_3_S_2_/coke solid waste (raw materials) utilizing KOH as the activator. The pore structure was modified using KCl as the template, leading to an enhanced distribution of Ni_3_S_2_. The hierarchical porous carbon produced a higher specific surface area of 2738 m^2^/g and a pore volume of 1.61cm^3^/g. The raw material, Ni_3_S_2_/PC, showed a clustered surface structure; however, after being etched with KOH, the surface of the porous carbons (Ni_3_S_2_/HPCs) roughened [96].

Pet coke was used to create porous AC (PAC), which was then activated with KOH to provide an effective anodic electrode material. In this case, the PAC was a practical contender for a supercapacitor electrode due to its BET surface area of 2105.6 m^2^/g and total pore volume of 0.941 cm^3^/g. The carbon atoms were layered and organized in a two-dimensional hexagonal pattern. Like an exfoliated graphene sheet, the PAC’s structure was a stacked layer of carbon atoms organized in a two-dimensional hexagonal pattern [97]. AC (AC-PHC and AC-PMC) was prepared by carbonization and direct KOH activation using pitch coke (PHC) and pet coke (PMC) as precursor materials. The structures of the two pieces of AC were amorphous. AC-PHC had a BET surface area of 2391 m^2^/g and a total pore volume of 1.15 cm^3^/g, while for AC-PMC, it was 1819 m^2^/g and 0.88 cm^3^/g. This suggested that PHC is more activation-sensitive to KOH [98].

Anode-grade pet coke with a higher volatile content is made of granulated AC. This experiment examined the effects of water vapor activation and pet coke carbonization. A maximal BET surface area approaches 400 m^2^/g with an AC production of up to 30 wt.% of the feedstock. The findings indicated that a standard carbonization temperature of 700 °C and subsequent activation in a water vapor environment at 800 °C are required for pet coke. These ideal thermal treatment conditions can be considered a standard for producing granulated AC [99]. Heat treatment was also carried out to obtain oxygen-rich AC using pet coke as a starting material. In contrast, it was accessible via KOH activation at a high heating rate. Comparing the ACs to the raw materials pet coke and carbonized pet coke (C-PC), the former showed more significant surface fractures and roughness. The AC sample AC5 yielded a maximum BET surface area of 3030 m^2^/g, along with micropore and mesopore volumes of 0.43 cm^3^/g and 1.45 cm^3^/g [100]. Because of their high power, capacitance, and safety, cylindrical supercapacitors are the perfect energy storage solution for electric vehicle applications. Researchers created AC sheets (ACS) from pet coke with a surface area of 2394 m^2^/g and pore volume of 1.44 cm^3^/g. They assessed the commercial viability of these sheets by creating a cylindrical supercapacitor with dimensions of 60 × 80 mm (D × H) and specifications of 2.7 V and 1200 F. A commercially available supercapacitor of comparable size was diffusely compared with the indigenous cylindrical device, and the supercapacitor developed in this study shows significant potential in terms of attractive electrochemical characteristics for practical use [101].

**Figure 4:**
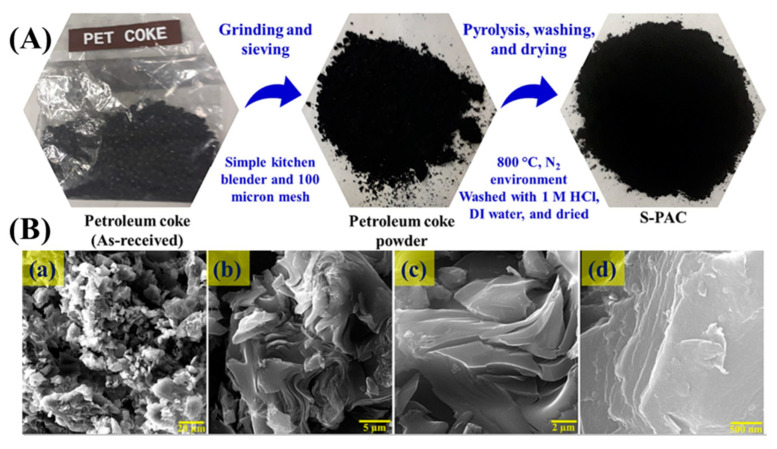
(**A**) Schematic representation from the S-PAC synthesis from the pet coke and (**B**) the corresponding (**a**–**d**) FESEM micrographs. Reproduced with permission [3]. Copyright 2023, Elsevier.

Shah et al. embarked on a pioneering study to enhance the efficacy and accessibility of supercapacitors, focusing on developing a novel electrode material derived from pet coke [3]. Their research heralds a significant breakthrough in energy storage, particularly in synthesizing AC with superior electrochemical properties. Unlike traditional biomass-derived AC, which suffers from low yield and high production costs due to its limited carbon content, using PC as a precursor offers a promising alternative due to its high carbon content of approximately 90 wt.%. This shift not only addresses the cost and efficiency challenges but also leverages the abundant availability of pet coke. The core of this work lies in their innovative approach to synthesizing sulfur-doped porous AC (S-PAC) from pet coke. The process begins with the mechanical crushing of PC to obtain a fine powder, which is then mixed with potassium bicarbonate (KHCO_3_) in a 1:4 ratio by mass, as shown in Figure 4A. This mixture undergoes a heat treatment at 800 °C for five hours in a nitrogen gas atmosphere, forming S-PAC. The final product is achieved after washing and drying steps, producing an AC with a remarkable yield of around 90%, significantly higher than its biomass-derived counterparts. Characterization techniques such as Brunauer–Emmett–Teller (BET) analysis, X-ray diffraction (XRD), and Raman spectroscopy reveal the amorphous structure and high porosity of S-PAC, with a BET surface area nearing 450 m^2^/g. This structural configuration is conducive to its application in supercapacitors, as it facilitates efficient ion transportation and storage. Furthermore, the XRD and Raman spectroscopic analyses underscore the material’s graphitic nature and defect-rich characteristics, essential for its electrochemical performance. The surface morphology, elucidated through field-emission scanning electron microscopy (FESEM), displays a heterogeneous mix of rough and smooth areas with an interconnected pore network, as shown in Figure 4B. This morphology is critical for the rapid diffusion of ions, thus enhancing the supercapacitor’s performance. X-ray photoelectron spectroscopy (XPS) analyses provide insight into the material’s chemical composition and functional groups, highlighting the presence of carbon, oxygen, and sulfur, with sulfur doping playing a pivotal role in improving the electrochemical properties. Electrochemical tests of the S-PAC-based supercapacitors, including cyclic voltammetry (CV), galvanostatic charge–discharge (GCD), and electrochemical impedance spectroscopy (EIS), demonstrate outstanding performance with a specific capacitance of approximately 140 F/g at a current density of 0.5 A/g. This high capacitance, coupled with excellent charge–discharge cycle stability, underscores the potential of S-PAC as a superior electrode material for supercapacitors. This research introduces a cost-effective and high-yield method for producing AC from petroleum coke and highlights S-PAC’s enhanced electrochemical performance. The study’s findings open new avenues for developing advanced supercapacitors, offering a promising solution to energy storage challenges and paving the way for future innovations.

A straightforward chemical activation process created ACs with a high specific surface area using semi-coke, a coal derivative obtained by dry distillation. The produced ACs had a pore capacity of around 1.5 cm^3^/g and a specific surface area of 2522 m^2^/g. The increase in surface area is noticeable with the rise in KOH amount during activation [102]. The treated ultra-fine needle coke particles (UNCs) were turned into hierarchical porous ultra-fine carbon fibers via electrospinning and thermal treatment procedure using two immiscible polymers such as polyacrylonitrile (PAN) and polymethylmethacrylate (PMMA). The rough ultra-fine carbon fibers of PAN, PMMA, and UNCs exhibited a high pore volume of 1.19 cm^3^/g and a high surface area of 919.3 m^2^/g [103]. Using KCl as an auxiliary template and KOH as a chemical activator, the refined solid waste (RSW) was utilized as the raw material to fabricate molybdenum sulfide/hierarchical porous carbon (MoS_x_/HPC) through the carbonization and activation process. With a mass ratio of 1:3:3, the MoS_x_/HPC produced with raw material/KOH/KCl has a large specific surface area (2908.9 m^2^/g), a total pore volume of 1.53 cm^3^/g, and a hierarchical micro-mesoporous structure [104]. 

Wasted bleaching earth carbon, SBE@C, was created via the pyrolysis of spent bleaching earth (SBE) collected from an oil refinery as a solid by-product. For SBE, the average pore width, volume, and specific surface area were 16.29 nm, 7.17 × 10^−2^ cm^3^/g, and 16.50 cm^2^/g, respectively. When SBE@C was compared to SBE, its average pore size dropped to 11.20 nm, indicating a typical mesoporous material, while its specific surface area and pore volume grew to 97.62 m^2^/g and 2.77 × 10^−1^ cm^3^/g, respectively [105]. Crumpled nitrogen-doped porous carbon (NPC) was produced by pyrolyzing petroleum pitch and employing urea as a pore-forming agent. Petroleum pitch is readily available and inexpensive. The NPC-2 porous carbon sample had a pore volume of 1.20 cm^3^/g and a surface area of 174.7 m^2^/g. To address the problem of electromagnetic radiation pollution, its effectiveness as an electromagnetic wave absorbing (EWA) material was assessed [106].

Four different forms of hydrocarbons, vacuum residue, asphalt, heavy catalytic gasoil, and heavy shale oil, each with different origins, compositions of group hydrocarbons, and properties, were employed to produce pet coke in a thorough investigation. A laboratory delayed coking unit with process parameters ranging from 1.5 to 3.5 mg at 500 °C was employed to carry out this study. At 750 °C, KOH was used to activate the generated coke chemically. The morphology of coke samples derived from raw materials that contain a significant quantity of resins and paraffin naphthenic fraction (vacuum residue and asphalt) was the same. Conversely, coke derived from highly aromatic residues (HCG) had surface agglomerates of smaller particles, leading to increased porosity. Compared to other process pressures, the largest specific surface area was found in carbon compounds formed at 1.5 atmg. The carbon from vacuum residues exhibited the largest BET surface area (2302 m^2^/g) [107]. A simple dual template technique created heteroatom-doped hierarchical porous carbon (h-PC) with nanosheets/hollow nanospheres multi-scale structure, utilizing petroleum asphalt as the carbon precursor. The ratio of target templates was changed to regulate the multi-scale pores. The morphologies of nanosheets and nanospheres were discovered to be present in the hybrid structure of the h-PC-1 and h-PC-2 materials. The carbon structure of micro-PC material was thick and resembled a honeycomb. The Meso-PC material exhibited a property of thin, crinkled carbon nanosheets [108].

Carbon materials from vacuum residue (a low-cost petroleum waste) were activated using KOH, MgO, and Ca(OH)_2_ at a comparatively lower 400 °C. Micron-sized particles in carbon samples treated with KOH, Ca(OH)_2_, and MgO had asymmetrical morphologies. The most significant total pore volume (5.3 cm^3^/g) and BET surface area (1250.6 m^2^/g) were found in KOH-activated vacuum residue-based carbon (VR-KOH) [109]. From fuel coke, nitrogen and phosphorus co-doped porous graphene sheets (a-NPGO) were created by impregnating the material with 0.5 mL of phosphoric acid and 0.6 mL of ethylenediamine and by annealing the material at 800 °C. The graphite oxide doped with phosphorus exhibited a highest surface area, measuring 420.4 m^2^/g, and a rough and uneven surface roughness [110].

A kind of non-nanoporous graphitic carbon was prepared via catalytic synthesis of porous carbon from coal, followed by graphitization, for the anode material of LIBs. By eliminating the micro/mesopores in the porous carbon, the graphitization process yielded a new type of carbon with extremely little porosity and flaws. This could avoid electrolyte side reactions, which are frequently observed in electrodes made of porous carbon materials. After annealing at 2500 °C, the specific surface area of the coal-based porous carbon (PC-900) was decreased to 32.5 m^2^/g (GC-2500). The graphitized non-porous sample had no micropores and mesopores, thus creating smooth surfaces [111]. In the limited area of the enlarged vermiculite, carbonization of the glucose(G) and coal tar pitch (CTP) molecules occurred, resulting in the production of distinct carbon nanosheets (G-CNSs and CTP-CNSs). While G-CNSs had very smooth dispersed fragments, indicating the presence of significant gaps between the layers, the surface of CTP-CNSs had numerous stacked layers. It was somewhat rough, resembling the structure of multi-layered graphene. The carbon nanosheets generated from coal tar pitches (CTP-CNSs) exhibited a total pore volume of 0.558 cm^3^/g and a specific surface area of 297 m^2^/g [112].

**Figure 5:**
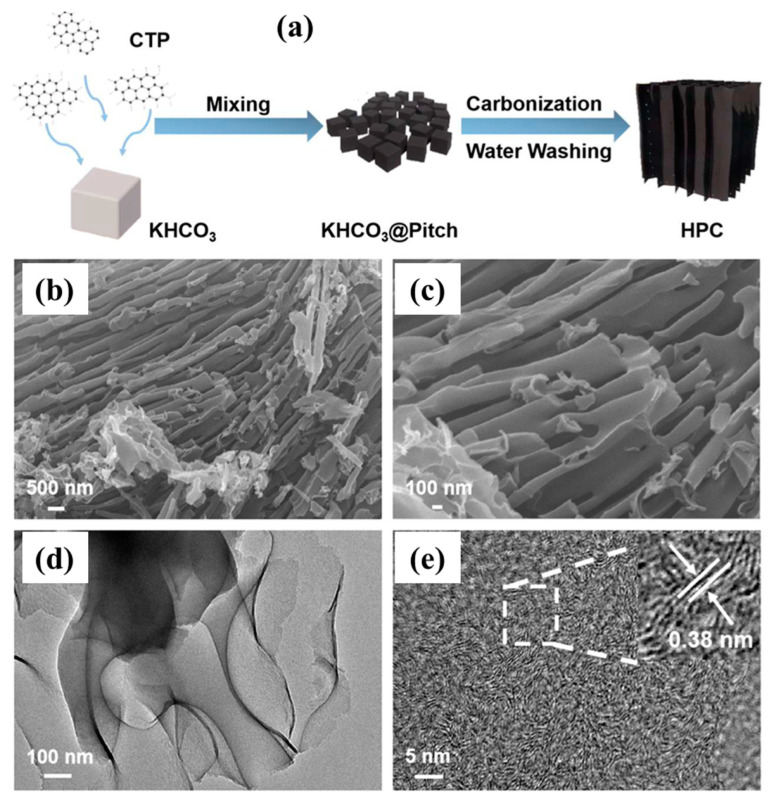
(**a**) Schematic representation for the synthetic protocols and (**b**,**c**) SEM images, (**d**) TEM image, and (**e**) HR-TEM image of HPC-3. Reproduced with permission [45]. Reproduced under the term CC-BY-4.0. Copyright 2023, Zhang et al. MDPI.

By incorporating NiCoP nanoparticles into the N, S co-doped porous carbon derived from pet coke (PCPC), sulfur-host PCPC/NiCoP composites were created. A multilayered structure was produced via carbonization and then activated with KOH. With the addition of KOH, the PCPC’s pores grew until reaching a maximum specific surface area of 471.20 m^2^/g. PCPC could effectively capture lithium polysulfides (LiPSs). This could be accommodated by the greater surface area and abandoned multistage holes of PCPC [113]. A green template technique was used to create hierarchically porous carbon (HPC) from coal tar pitch (CTP) and use it as the anode material for LIBs [45], as illustrated in Figure 5a. It was shown that the microstructure and electrochemical performances of HPC were highly influenced by the carbon source (CTP) mass ratio to the template (KHCO_3_). The HPC-3 synthesized at a 3:1 mass ratio exhibited a coral-like lamellar nanostructure (Figure 5b,c) with a specific surface area of up to 1643 m^2^/g and a pore volume of up to 0.99 cm^3^/g, effectively expanding the electrode–electrolyte contact interface and accelerating the lithium-ion diffusion kinetics [45]. Nanopores were also observed on the surfaces of the porous carbons, which was advantageous for the flow of electrolytes and provided enhanced ion transportation (Figure 5d). It also ensured better adsorption of lithium ions. A high-resolution TEM image also confirmed the presence of ordered lattice fringes. They remained in range with a lattice separating distance of 0.38 nm (Figure 5e).

Through a straightforward pickling procedure, waste coal-based filter materials were recycled into carbon anode materials for potassium and sodium-ion batteries. A mixed acid treatment removed contaminants from the waste filter material without damaging the nano graphite-like domain. With a maximum surface area of 35.4 cm^2^/g, the coal waste-based filter carbon (WFC-1400) was annealed at 1400 °C. Higher annealing temperatures were discovered to encourage the growth of layers resembling nano graphite [114]. Meso carbon microbeads (MCMB) were created by co-carbonizing the conductive carbon black (CCB) and fluid catalytic cracking (FCC) low-value decant oil [115]. By promoting nucleation, preventing mesophase sphere development, and adhering to sphere surfaces, CCB increased the yields (38.9 wt.%), which affected the microstructure of MCMB. Thus, ensuring an improved electrochemical performance. The BET surface area and total pore volume (18.2 m^2^/g and 0.06 cm^3^/g) were most significant in CMCMB-5.

**Table 1:** Summary of the preparation of electrode materials used in supercapacitor applications.

Carbon Materials	Carbon Source	Preparation Method	Method of Activation	Morphology	Surface Area (m^2^/g)	Total Pore Volume (cm^3^/g)	Specific Capacitance (F/g), Current Density (A/g)	Ref.
AC	Pet coke	pyrolysis	HCl	rough, fractured, and smooth surfaces, porous	450	0.08	140, 0.5	[3]
Hierarchically porous carbon	Coal tar pitch	pyrolysis	KHCO_3_	coral-like laminar structure	1643	0.99	660 mAh/g, 1	[45]
N, S-CLPACF	Coal liquefaction residue/polyacrylonitrile/terephthalic acid composite	pyrolysis	…	rough crosslinked nonwoven nanofiber	589	0.280	267, 0.2	[77]
AC	High-ash coal liquefaction residue	pyrolysis	KOH	irregular coral-type	1787.2	…	250, 1	[78]
Nitrogen-doped porous carbon (CNPCs)	Anthracite coal	pyrolysis	…	folded carbon nanosheet	1673.5	0.77	750 mAh/g, 0.1	[80]
AC	Coal liquefaction residue	pyrolysis	KOH	similar to tremella with internal overlapping, loose reticulated structure	3102	…	457, 0.5	[81]
Porous carbon	Coal-based green needle coke	pyrolysis	KOH	sheet-like streamline structure	807.7	0.370	274.9, 1	[82]
AC	Low-quality sub-bituminous coal	pyrolysis	KOH and KOH + NaOH	slightly crystalline and amorphous	789.910	0.103	57.63, 20 mV/s	[84]
AC	Anthracite coal	pyrolysis	KOH	uniformly distributed honeycomb pores	1207	1.346	243.6, 0.5	[86]
Hierarchical porous carbon	Coal and rice husk	pyrolysis	…	inter-connected hierarchical porous	…	…	352, 1	[88]
AC Graphitized carbon	Anthracite coal	pyrolysis	KOH	crystalline	2256.0 18.7	1.21 0.07	568.1 mAh/g, 0.1	[90]
High-value capacitive carbon	Coal-coke	pyrolysis	KOH	rough	1633	…	237, 0.2	[91]
AC	Pet coke	pyrolysis	KOH	sheetlike	1952	1.1	145, 0.1	[92]
Sulfur-doped porous carbon composite	Pet coke	pyrolysis	KOH	porous composite with nanowires	3318	1.67	327, 1	[93]
Carbon nano-onions (CNOs)	Pet coke	pyrolysis	KOH	sponge-like morphology	2665.5	1.2	312, 1	[94]
Multi-heteroatom self-doped graphene	Pet coke	sonication	…	amorphous	…	…	44, 0.5	[95]
Ni3S2/HPCs	Pet coke	pyrolysis	KOH	rough	2738	1.61	360, 1	[96]
PAC	Pet coke	pyrolysis	KOH	crystal	2105.6	0.941	470, 0.5	[97]
AC	Pitch coke and pet coke	pyrolysis	KOH	amorphous structure	2391 1819	1.15 0.88	146.4 mAh/g, 0.1	[98]
AC	Pet coke	pyrolysis	KOH	nanosheets	2394	1.44	128@1	[101]
AC	Semi coke	pyrolysis	KOH	amorphous	2522	1.5	310, 1	[102]
AC	Treated ultra-fine needle coke particles (UNCs)	pyrolysis	CO_2_	rough and hollow fibrous	919.3	0.37	387.2, 0.5	[103]
MoS_x_/ HPC-133	Refinery solid waste	pyrolysis	KCl and KOH	rough	2908.9	1.53	349.7, 1	[104]
Hierarchical porous carbon	Petroleum asphalt	pyrolysis	potassium citrate	thick and honeycomb-like carbon structure with thin and crumpled carbon nanosheets	1574	1.06	437, 1	[108]
Active C=carbon	Vacuum residue (VR)	pyrolysis	KOH	irregular shapes of micron-sized particles	1250.6	5.3	91.91, 0.5	[109]
Porous graphene	Fuel coke	pyrolysis	…	heterogeneous and rough surface	405.1	…	322, 1	[110]
PCPC/NiCoP/S composite	Pet coke	pyrolysis	KOH	multilayered graphene-like nanosheets	2471.20	…	1462.7 mAh/g, 0.1 C	[113]
AC	Anthracite coal	pyrolysis	KOH	amorphous	3550.7	2.168	280@0.5	[116]
AC	Coal gasification fine ash	pyrolysis	KOH	rough and significantly augmented pore structure, amorphous	2167.5	1.66	220@1	[117]
Mn/N-APC	Pet coke	pyrolysis	KOH	…	…	…	76.1, 0.1	[118]
Coal-derived carbon	Bituminous coal	pyrolysis	…	low crystalline, amorphous carbon	272.97	0.0249	270.1 mAh/g, 0.1 C	[119]
Graphitic carbon	Coal	pyrolysis	…	crystalline	372	0.52	262.2 mAh/g, 0.1C	[120]
Hierarchical porous carbon (HPC)	Coal tar pitch	pyrolysis	KOH	irregular dense carbon	1388.62	0.61	329, 1	[121]
Nanoporous carbon	Bitumen	pyrolysis	KOH	flaky appearance due to sheet-like structures	2117	1.1	380, 1	[122]
Carbon composite	Asphaltene	pyrolysis	KOH	honeycomb-like structure with spherical macropores	2264.6	1.246	112, 0.4	[123]

### 3.2. Performance of Carbon Materials as Supercapacitor Electrodes

The HPCs showed significant electrochemical performances due to the increased surface area and pore volume, which was validated by various electrochemical analyses of the samples. These HPCs were synthesized from coal tar pitch, assisted by CaO (as a hard template) via one-step carbonization and KOH activation. With an energy density of 6.45 Wh/kg and a power density of 483.7 W/kg, the symmetrical supercapacitor built with the HPCs-0.5-700-2 electrode demonstrated the maximum specific capacitance of 198 F/g at 1 A/g. A high capacitance retention of 66.4% was shown by the HPCs-0.5-700-2 supercapacitor, which had a specific capacitance of 198 F/g at 1 A/g and 132 F/g at 20 A/g. The HPCs-0.5-600-2 supercapacitor had a specific capacitance of 195 F/g at 1 A/g and a matching capacitance retention of 27.8% at 20 A/g. At a power density of 473.1 W/kg, HPCs-0.5-600-2 could deliver an energy density of 6.07 Wh/kg. After 4000 cycles, the HPCs-0.5-600-2 supercapacitor’s cyclic stability held steady at 56.9% of its original capacitance [121]. The KOH-activated, anthracite-based PAC showed a high specific capacitance of 280 F/g, an energy density of 38.9 Wh/kg at 0.5 A/g, and a maximum power density of 20,000 W/kg at an energy density of 27.8 Wh/kg when used as an electrode for a symmetric capacitor. Due to the unique structure, large specific surface area, and porous nature, the capacity exhibits excellent cyclic behavior, with nearly no deterioration seen after 30,000 cycles at varying current densities [116]. 

Coal gasification fine ash-derived porous carbon electrode provided an excellent life of 15,000 cycles. The symmetrical supercapacitor’s capacitance retention rate was 97.1%, and its energy density was 13.4 Wh/kg at a power density of 475 W/kg. The device’s mass-specific capacitance (Cs) was shown to be 107.16 F/g at a current density of 0.5 A/g [117]. The effect of nitrogen and sulfur doping on cross-linked Preasphaltene-based carbon nanofiber was evident in the electrochemical performance of the N, S-CLPACFs. Compared to the results obtained with PACF (133 F/g) and CLPACF (155 F/g), the capacity of N, S-CLPACF was 170 F/g at 0.2 A/g. N, S-CLPACF outperformed PACF (64%), demonstrating 71% capacitance retention with an increase in current density from 0.2 to 100 A/g. The results indicated that the large SSA, coexistence of N and S heteroatoms, and cross-linked structure mainly caused the large specific capacity and high-rate capacity. N, S-CLPACF exhibited the highest energy density of 4.7 Wh/kg at a power density of 1.2 kW/kg. It also showed excellent long-term cyclic stability, as evidenced by its capacitance retention of 98% after 10,000 cycles at 2 A/g [77].

Electrochemical characteristics of HPCs obtained from the carbonization of ashes of CLR were assessed at a potential range of 0 to 1.0 V in a 6 M KOH aqueous solution by a two-electrode setup. For HPC-600, the specific capacitance at a current density of 0.5 A/g was 197 F/g, with an energy density of 7.1 Wh/kg at a power density of 62.5 W/kg. Following 10,000 charging and discharging cycles, 99.7% of the capacitance was retained in the HPCs [78]. Anthracite-based nitrogen-doped porous carbon materials exhibited noticeable changes in specific areas and electrochemical properties as the amount of g-C_3_N_4_ was used as a template during preparation. At a current density of 0.1 A/g, the highest specific capacitance of a symmetrical capacitor (CTK-1.0//CTK-1.0) was found to be 56.1 F/g. The energy density was 71.8 Wh/kg at a high-power density of 20.5 kW/kg. After 3000 cycles, they showed a high-capacity ratio of 75% at a current density of 2 A/g. This indicated their outstanding cyclic stability [80].

An outstanding cycling life was demonstrated by the hierarchical porous carbons’ (OHPCs) electrode-built symmetric supercapacitors, with 93% retention over 10,000 cycles at 4 A/g. Oxygen self-doped hierarchical porous carbons (OHPCs) were derived from CLR in this study. With a power density of 10,000 W/kg, this device yielded an impressive energy density of 35.2 Wh/kg and 46.5 Wh/kg when the aqueous electrolyte was substituted with an organic or ionic liquid. At 5 4 A/g, the OHPC-800 demonstrated the maximum specific capacitance of 160 F/g [81]. The optimal ideal electric double-layer capacitive performance was achieved by thermally treating synthesized porous carbon from coal-based green needle coke at 750 °C, which is superior to those treated at other temperatures. A symmetric button-type supercapacitor was built using the porous carbon supported on Ni-foam as both the positive and negative electrodes. With a power density of 1031.42 W/kg, the device yielded a notable energy density of 20.51 Wh/kg, a capacitive retention rate of 75.8% at 12 A/g, and a high capacitance of 147.7 F/g. Remarkably, after 5000 cycles at 1 A/g, the built-in supercapacitor exhibited exceptional cycling stability, with a capacity retention of 95.6% and a Coulombic efficiency of 98.5% [82].

In 1-M tetraethylammonium tetrafluoroborate (TEABF_4_) in association with Acetonitrile (ACN) in a two-electrode system, the AC (JCRA2) permitted an excellent power density of 2460 W/kg with a high potential window of 2.5 V, and the highest power density of 2592 × 10^4^ W/kg with a 1.2-V potential window in 6-M KOH. The supercapacitor cell exhibited outstanding cyclic stability over a thousand cycles during charging and discharging. With a maximum power density of 13,775.99 W/kg and a specific capacitance value of 7 F/g, the ACs in 1-M TEABF_4_ in ACN exhibited robust cyclic stability for over 1000 cycles, making them suitable for use in tiny and general electronics [84]. JCRA2’s porous structure and large specific surface area allowed for better-connected transport channels between the electrode and electrolyte ions, increasing both the specific capacitance and the area of the Cyclic Volumetry (CV) curve [124]. The electrode material prepared from coal-based carbon dots showed enhanced electrochemical performances due to many surface-active sites and higher surface area. At a current density of 20 A/g, the electrode from coal-derived AC carbonized at 800 °C (CDAC-800) had a specific capacitance of 191.6 F/g, and after 10,000 cycles, it maintained around 98.1% of its capacitance, demonstrating its remarkable cycling stability. In addition, this electrode material’s highest specific capacitance value was 243.6 F/g at a current density of 0.5 A/g [86].

The combined application of rice husk and coal was shown in a study where the findings showed that an electrode fabricated from raw materials using a rice husk-to-coal ratio of 3:1 (HPC-RH_3/1_) exhibited a capacitance of 268 F/g at 20 A/g in a three-electrode setup in an electrochemical analysis. On the other hand, at 500 W/kg of power density, this demonstrated an energy density of 6.98 Wh/kg in a two-electrode symmetric supercapacitor system. Surprisingly, after 10,000 cycles, HPC-RH_3/1_’s stability remained at 100%, and the distribution of its C, O, and N components was notably stable [88]. The pre-lithiation of AC covered with graphene (AC4@5rGO) performed better than non-coated graphitic oxide and AC. According to electrochemical tests, it had an ultrahigh specific capacitance of up to 302 F/g and a respectable capacity retention of 75% after 10,000 cycles. With a reversible specific capacity of 568.1 mAh/g at 0.1 A/g, the graphitic carbon anode coated with graphene (GC5@1rGO) maintained 97% of its capacity after 300 cycles. High energy density, power density (315.1 Wh/kg at 300 W/kg and 133.3 Wh/kg at 15,000 W/kg), and improved cyclic stability (78% capacity retention after 10,000 cycles) were achieved by the optimized AC4@5rGO/prelithiated GC5@1rGO LIC [90].

**Figure 6:**
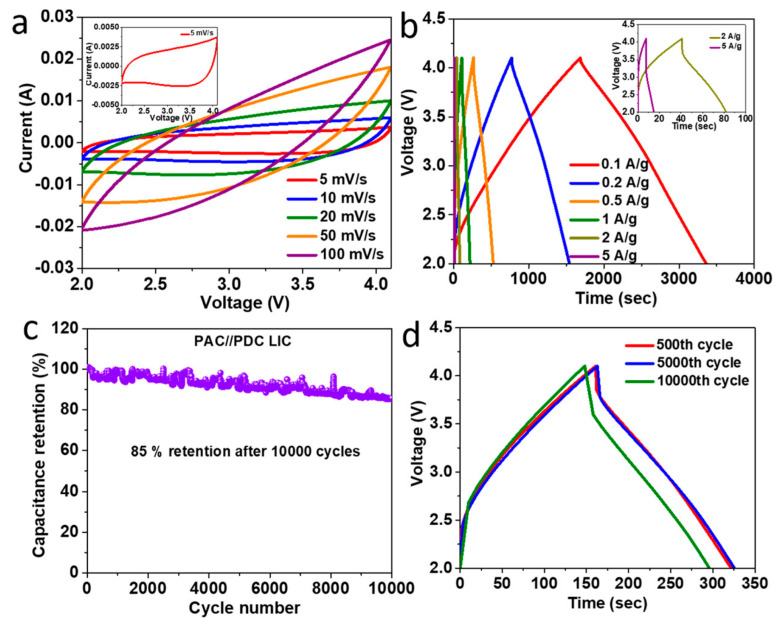
Electrochemical performance of LIC with pet-coke-derived carbons: (**a**) cyclic voltammetry, (**b**) charge–discharge at various loads, (**c**) cyclic stability, and (**d**) long-term charge–discharge behavior. Reproduced with permission [92]. Copyright 2021, American Chemical Society.

Electrochemical experiments by Gao et al. demonstrated that electric double-layer and Faradic processes coexist in coal-carbon, resulting in a high capacitance of 237 F/g at 0.2 A/g, which is twice as high as commercial active carbons. Moreover, after 3000 cycles of continuous current charge and discharge, its capacitance performance stayed at 92% [91]. A lithium-ion capacitor with a long lifespan (85% capacity retention after 10,000 charge–discharge cycles at 1 A/g) was created using carbon materials generated entirely from pet coke. It also showed a high energy density of 80 Wh/kg and a high power density of 8.4 kW/kg [92]. Figure 6 presents various electrochemical tests conducted on an LIC made with carbon materials derived from pet coke. The shape of the curves indicates capacitive behavior with higher currents at higher scan rates. The inset shows a detailed view of the voltammogram at a five mV/s scan rate. The shape charge–discharge profiles represent the LIC’s efficiency and capacity at each current density, with the inset showing how the device performs under higher loads (2 and 5 A/g). The graph shows the long-term cyclic stability of the LIC, plotting the percentage of capacitance retention over 10,000 cycles. The device retains 85% of its initial capacitance after extensive cycling, indicating good durability. The charge–discharge profiles at selected cycles (500th, 5000th, and 10,000th) at a constant current density of 1 A/g demonstrate how the LIC’s performance evolves over time. Although MnO_2_ reduced the surface area and pore volume of HPCs derived from pet coke, the electrochemical activities improved significantly. The specific capacitance of the asymmetric battery (HPC//HPC/MnO_2_) at a current density of 0.5 A/g with 1 M Na_2_SO_4_ as the electrolyte was 110.4 F/g. Following five thousand cycles, the symmetric battery (HPC//HPC) maintained a capacitance retention rate of 96.5%, whereas HPC//HPC/MnO_2_ maintained a rate of 79.1%. At 450 W/kg, the energy density of HPC//HPC/MnO_2_ was 49.7 Wh/kg; however, for HPC//HPC, it was 29.6 Wh/kg [93]. Carbon nano-onions (CNOs) prepared from pet coke and coated with coal tar pitch exhibited superior electrochemical activity due to its highly curved multilayered shell structure. The specific capacitance of a pitch-coated CNO (P@PC-AC)-based electrode in a two-electrode symmetrical supercapacitor (SSC) (P@PC-AC//P@PC-AC) was 40.8 F/g for current densities of 1 and 20 A/g, respectively. P@PC-AC in an aqueous electrolyte provided an excellent energy density of 7.47 Wh/kg at a power density of 221 W/kg and held onto 3.6 Wh/kg at a power density of 6749 W/kg. At a current density of 5 A/g, 94.4% of the original capacitance was preserved after 10,000 cycles [94].

The pet coke-fabricated graphene-based device also demonstrates high Coulombic efficiency and cycle stability. The capacitance retention after 3000 cycles was ~98.78%. The manufactured symmetric supercapacitor displayed a maximum specific capacitance of 44 F/g at 0.5 A/g current density with a good energy density of ~8.8 Wh/Kg coupled with a power density of ~800 W/Kg at 0.5 A/g current density. [95]. Ni_3_S_2_/coke solid-waste-derived hierarchical porous carbon (Ni_3_S_2_/HPC) was tested for its electrochemical characteristics in a symmetric electrode assembly and three-electrode system. The single electrode capacitance of the capacitor in the symmetric electrode assembly was 43.67 F/g at 1 A/g. Still, in the three-electrode system, the capacitance of Ni_3_S_2_/HPC-3 reached 360 F/g at a current density of 1 A/g, which was a much higher value. The symmetrical supercapacitor achieved a power density of 19.65 Wh/kg at 450 W/kg in a 1 M Na_2_SO_4_ electrolyte, outperforming most previous carbon-based symmetrical supercapacitors [96]. Over a potential window of 0 to −1 V, the PAC prepared from pet coke provided a specific capacitance of 470 F/g at a current density of 0.5 A/g. It demonstrated a remarkably steady electrode performance with 100% Coulombic efficiency and 98% capacitance retention even after 15,000 GCD cycles [97].

The specific capacities of ACs from pitch coke (AC-PHC) and pet coke (AC-PMC) were 146.4 and 88.8 mAh/g, respectively, with a current density of 0.1 A/g. The resultant specific capacitance was due to good oxygen functional groups, rich surface area, and adequate porosity. The morphologies and physical characteristics (surface area and pore volume) proved that the effect of KOH activation is much higher on pitch coke-based AC. At 10 A/g, the AC-PHC electrode exhibited excellent retention of 85.0 mAh/g. It is also possible to obtain an exceptional power output of 15.8 kW/kg and a decent energy production of 117 Wh/kg. After 5000 cycles at 5 A/g, the capacity retention might reach up to 95%, indicating the remarkable cyclic stability of the zinc ion hybrid supercapacitors based on AC-PHC [98]. When tested in a coin cell application, the electrode fabricated using pet-coke-derived AC sheets demonstrated specific capacitance (128 F/g at 1 A/g) of super capacitive level. In a system consisting of three electrodes with 6M KOH electrolyte, it had a specific capacitance of 269 F/g at a current density of 1 A/g and kept a high capacitance of 205 F/g at a very high current rate (20 A/g) with an excellent capacitance retention of 76%. The symmetric supercapacitor demonstrated exceptional qualities of AC sheets as an electrode material with a high-power density of 32,800 W/kg and a high energy density of 20.2 Wh/kg. The ACS electrode’s long-lasting electrochemical property was ascertained by conducting cyclic stability tests at 1 A/g for over 20,000 cycles. The electrode exhibited high Coulombic efficiency and maintained over 91% of its original capacitance [101].

**Figure 7:**
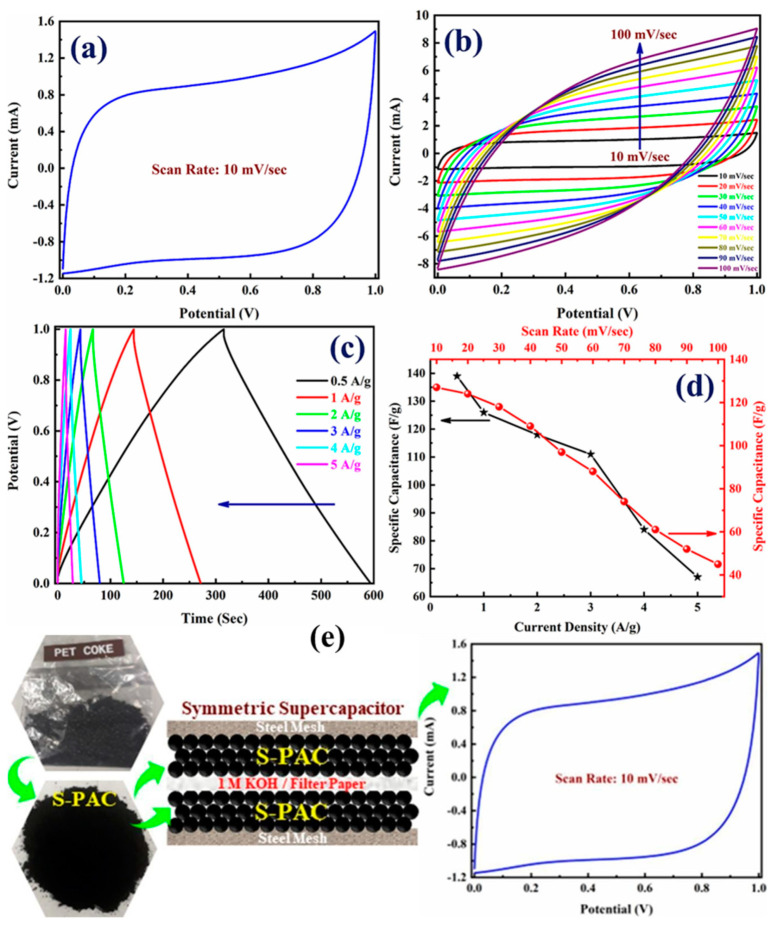
The S-PAC-based supercapacitor showcases (**a**) CV curve obtained at a scan rate of 10 mV/s, (**b**) CV curves at varying scan rates, (**c**) GCD profiles at assorted current densities, and (**d**) the variation in specific capacitance concerning different current densities and scan rates. (**e**) A schematic representation for the supercapacitor cell using S-PAC as electrode materials. Reproduced with permission [3]. Copyright 2023, Elsevier.

Shah et al.’s comprehensive study introduces a groundbreaking approach to supercapacitor development, employing S-PAC derived from pet coke as an electrode material [3]. This novel material stands out due to its high carbon content, cost-effectiveness, and exceptional electrochemical performance, making it an ideal candidate for energy storage applications, particularly in supercapacitors. The adaptability of S-PAC to wide operating potential windows (OPWs) is a hallmark of its design, which is crucial for enhancing supercapacitor performance. Figure 7 offers a comprehensive view of the electrochemical performance of the S-PAC when utilized in a supercapacitor. CV curve analysis demonstrated that S-PAC maintains its quasi-rectangular shape across various OPWs (0 to 1 V), indicating its robustness and efficiency in energy storage and retrieval. This behavior underscores the electrode material’s capacitance retention ability, even when subjected to different voltage ranges, making it versatile for various applications. Furthermore, the cyclic stability of the S-PAC-based supercapacitor is remarkable, maintaining approximately 87% Coulombic efficiency and retaining about 90% of its initial capacitance after 1000 GCD cycles at a current density of 10 A/g. This durability is crucial for practical applications, ensuring long-term reliability and consistency. The minor decline in capacitance is attributed to structural changes and electrolyte ion trapping, which is a common challenge in supercapacitors that have managed to minimize effectively. Shah et al. also explored the energy and power densities of the S-PAC-based supercapacitor, achieving a commendable balance between the two. The Ragone plot demonstrates that the supercapacitor can deliver an energy density of about 20 Wh/kg at a power density of 250 W/kg, with a reduced energy density at higher power, maintaining performance efficiency across various operational demands. The research offers a profound contribution to energy storage, showcasing the potential of S-PAC as a high-yield, cost-effective, and efficient electrode material for supercapacitors. Their work paves the way for future advancements in supercapacitor technology, possibly integrating nanostructured materials to enhance capacitance and energy density further. This study marks a significant step towards developing sustainable, high-performance energy storage solutions, aligning with the global shift towards renewable energy and the need for reliable, efficient energy storage systems.

**Figure 8:**
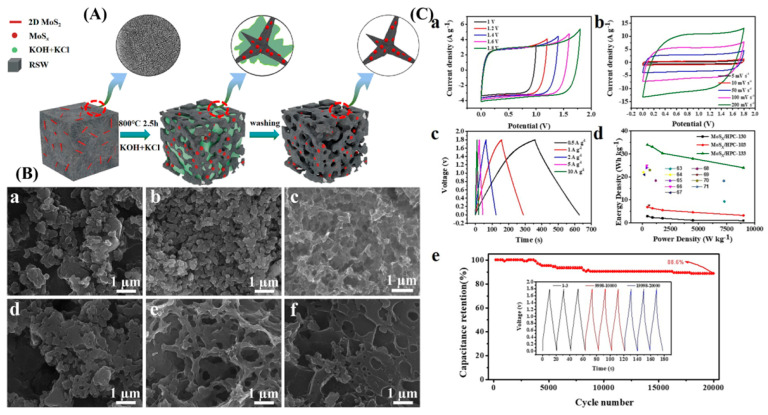
(**A**) Schematic representation for MoS_x_/HPC composites preparation. (**B**) SEM images of (**a**) raw RSW, (**b**) MoS_x_/HPC-130, (**c**) MoS_x_/HPC-103, (**d**) MoS_x_/HPC-113, (**e**) MoS_x_/HPC-133, and (**f**) MoS_x_/HPC-153. (**C**) The electrochemical performance evaluations of the MoS_x_/HPC-133 symmetrical supercapacitor: (**a**) CV profiles at various OPWs with a uniform scan rate of 50 mV/s, (**b**) CV profiles over a broad scan rate spectrum from 5 to 200 mV/s, (**c**) GCD profiles at a range of current densities from 0.5 to 10 A/g, (**d**) Ragone plots comparing the energy and power densities of the MoS_x_/HPC symmetrical supercapacitor against other documented carbon-based supercapacitors, and (**e**) cycling stability of the supercapacitor in a 1 M Na_2_SO_4_ electrolyte. Reproduced with permission [104]. Copyright 2023, Elsevier.

The semi-coke-produced AC-based supercapacitors demonstrated a remarkable specific capacity of 301 F/g at a current density of 1 A/g, and after 10,000 charge/discharge tests, it had a capacity retention of 95.9%. The samples’ surface area was observed to grow as the potassium hydroxide level rose, and the surface displayed an interconnected pore structure. The carbon material with the linked pore structure acts as a route for charge transmission, a prominent feature of materials suitable for the electrochemical double-layer capacitor [102]. Hierarchical porous ultra-fine carbon fibers were synthesized using ultra-fine needle coke particles associated with polyacrylonitrile and polymethyl methacrylate. At 1 A/g, the symmetric supercapacitor with the hierarchical porous produced electrode (PMNC4) showed an exceptional capacitance of 191.7 F/g. After 10,000 cycles, the specific capacity of the supercapacitor based on the PMNC4 electrode only decreased by 2.5% relative to its starting value. Furthermore, after 10,000 cycles, the Coulombic efficiency maintained a 99.4% level. At a power density of 489 W/kg, it demonstrated a notable energy density of 27.87 Wh/kg [103]. Refined-solid-waste (RSW)-derived hierarchical porous carbon (MoS_x_/HPC) was analyzed for electrochemical performance, as shown in Figure 8A [104]. This study clearly showed the activation and porous structures’ development paths. The results obtained from the SEM image illustrate the activation’s effects on the pure RSW. Previously, it was amorphous with no pores, with an average diameter of nearly 1.0–5.0 µm (Figure 8(Ba)). When KCl was applied as an activation agent for this material, the morphology almost remained intact. However, it decreased average particle sizes, shrinking them to below 1.0 µm (Figure 8(Bb)). On the other hand, the utilization of KOH turned out to be effective in activating MoS_x_/HPC nanocomposites. The morphology of this material became rougher with the application of KOH, keeping the average particle size within 0.3–1.0 µm (Figure 8(Bc)). The increased amount of KCl significantly contributed to the development of the porous structure of the nanocomposite (Figure 8(Bd)). Initially, MoS_x_/HPC-113 samples exhibited larger particles but relatively tiny pores. In MoS_x_/HPC-133, a highly porous structure was observed when the portion of KCl was increased (Figure 8(Be)). Figure 8C illustrates the electrochemical characteristics of the MoS_x_/HPC-133 symmetrical supercapacitor. The results collectively showcase the performance metrics of the MoS_x_/HPC-133 symmetrical supercapacitor, from its electrochemical stability and responsiveness to varying operational conditions to its energy storage efficiency and durability. The CV curves across different voltages and scan rates reveal the supercapacitor’s robust capacitive behavior and adaptability. GCD curves across a spectrum of current densities illustrate the device’s practical energy storage and discharge capabilities, while the Ragone plots offer a comparative perspective on its energy and power density performance. Lastly, the cycling stability test underscores the supercapacitor’s long-term reliability and operational stability, marking it a promising candidate for energy storage applications. During the electrochemical analysis, at 0.5 A/g, the specific capacitance of a symmetric capacitor was 75.5 F/g. The constructed supercapacitors exhibited exceptional electrochemical activity with a high power density of 450.03 W/kg and an energy density of 33.99 Wh/kg in a 1 M Na_2_SO_4_ aqueous electrolyte. After 20,000 cycles, the MoS_x_/HPC-133//MoS_x_/HPC-133 symmetric supercapacitor showed exceptional cyclic stability, maintaining 88.6% of the original capacitance.

Petroleum asphalt-derived heteroatom-doped hierarchical porous carbon (h-PC) exhibited a hollow multiscale structure that effectively served in a symmetric and solid-state capacitor system. In a symmetric supercapacitor system with 6 M KOH as an electrolyte, h-PC-2 demonstrated an exceptional rate performance with a specific capacitance of 93.3 F/g at 1 A/g. At 10 A/g, the Coulombic efficiency stood at 100%, and the capacitance retention reached 92.6% after 20,000 charge–discharge cycles. The energy density was increased to 25.5 Wh/kg at 450 W/kg while employing Na_2_SO_4_ electrolyte. h-PC-1’s SSA was estimated to be 1574 m^2^/g, more than Meso-PC’s (161 m^2^/g) and Micro-PC’s (1243 m^2^/g) values. Solid-state capacitors prepared using h-PC demonstrated a maximum energy density of 12.95 Wh/kg at a power density of 250 W/kg [108].

Using Mg(OH)_2_ nanoplates as the template and in situ KOH activation, nanoporous carbon was synthesized from bitumen, hexane-insoluble asphaltenes, and N, N-dimethylformamide (DMF)-fractionated asphaltenes. The purpose was to evaluate its performance as an electrode material for electrochemical storage applications. However, the NiO in the nanoporous NiO/carbon composite behaves pseudocapacitive. Thus, asphaltene-derived NiO/C composite electrodes show increased capacitance. The maximum capacitance was found in the nanoporous carbon derived from hexane-precipitated asphaltenes (ACNS1), even though BCNS (nanoporous carbon from bitumen) had the biggest specific surface area (2117 m^2^/g). In total, 380 F/g was the gravimetric capacitance of ACNS1 determined by GCD measurement at 1 A/g. The capacitances of BCNS, ACNS2, and the reduced graphene oxide (rGO) were considerably lower than ACNS1 and ACNS3. The ACNS1 electrode had high cyclic stability, as demonstrated by about 85% capacitance retention at a current density of 5 A/g after 1000 cycles [122]. Three activating agents (KOH, MgO, and Ca(OH)_2_) were used to prepare vacuum residue-based AC materials for electrochemical usage. With 91.91 F/g, it also showed the highest capacitance. The findings demonstrated that VR-Ca(OH)2 and VR-MgO capacitance exhibited comparable values when adding carbon black. In contrast, the capacitance of VR-KOH increased by only around 50%, suggesting that macropores were the primary source of capacitance [109].

**Figure 9:**
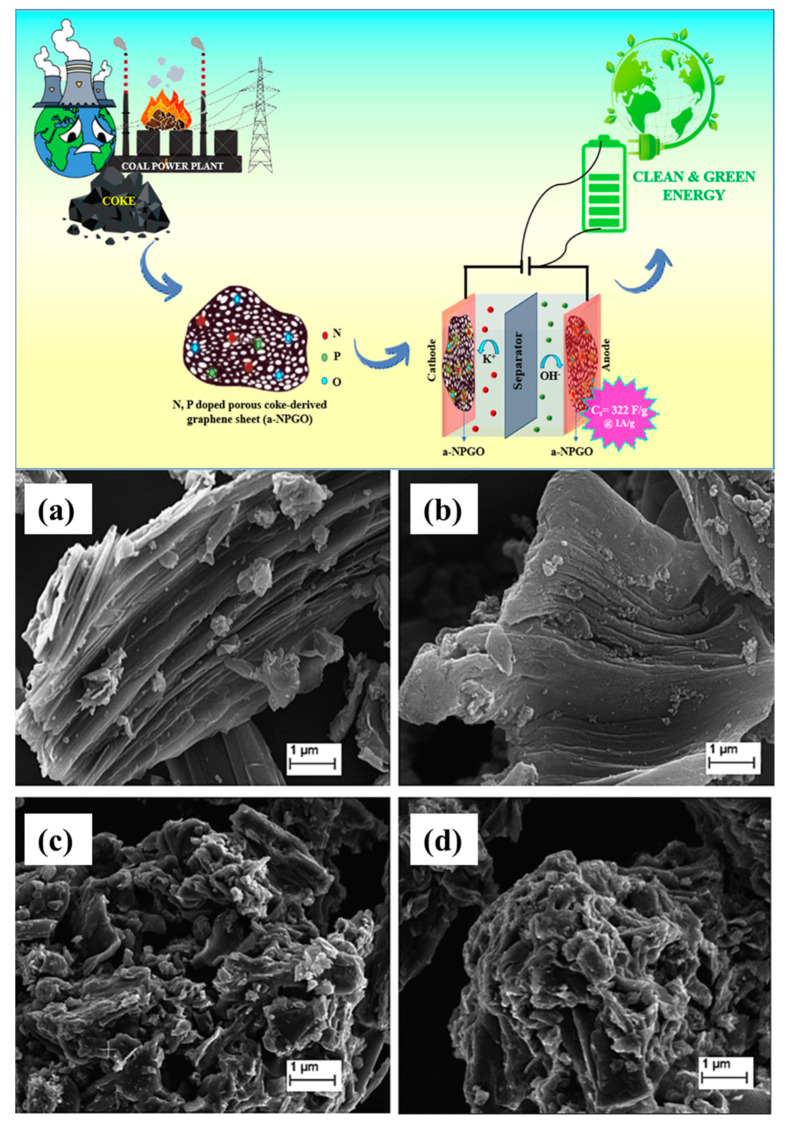
Nitrogen and phosphorus co-doped porous graphene structures derived from fuel coke for high-performance supercapacitors. SEM image illustrating the surface structure of (**a**) pristine GO, (**b**) hetero atom-doped GO, along with activated (**c**) a-PGO and (**d**) a-NPGO graphene sheets. Reproduced with permission [110]. Copyright 2023, Elsevier.

Asphaltene was effectively converted into carbon electrode materials using a simple thermal method involving KOH activation and melamine foam as a template. The greatest BET surface area (2264.6 m^2^/g) and total pore volume (1.246 cm^3^/g) were found in the carbon material (5:1)-0.33 h-900, which was made with a KOH to asphaltene ratio of 5:1, at a homogenization time of 20 min and thermal treatment temperature of 900 °C. On the other hand, the optimized material (3:1)-1 h-700-0.5 h with a homogenization time of 1 h and heat treatment at 700 °C attained a maximum specific capacitance of 112 F/g at a current density of 0.4 A/g. The symmetric supercapacitor’s specific energy and specific power were 8.6 Wh/kg and 645 W/kg, respectively. It was discovered that heat treatment at temperatures exceeding 800 °C resulted in the broken carbonaceous structure, further decreasing the number of heteroatom-based functional groups previously derived from the heteroatoms. Alongside this, the degree of graphitization was also reduced. These functional groups, resulting from asphaltene or melamine, were responsible for the desired pseudo-capacitive action. It was also proposed that one of the principal mechanisms for developing enhanced specific surface area was the homogeneity of the emulsion containing the carbon source and the activating agent. To produce smaller microdroplets, it was, therefore, essential to employ high stirring speeds or to extend the homogenization period to generate a hierarchical design [123]. Nitrogen and phosphorus co-doped porous graphene sheets (a-NPGO) were created from fuel coke via impregnation and annealing at 800 °C. In two-electrode systems, the a-NPGO-modified electrode showed a specific capacitance of 167 F/g at a current density of 1 A/g. The a-NPGO-constructed supercapacitor demonstrated great cyclic stability of 90% after 5000 cycles, extraordinary power density of 812 W/kg at an energy density of 14 Wh/kg, and decreased charge transfer resistance of 2.8 Ω. All these proved its enormous potential as an electrode material for supercapacitors with superior performance [110]. The configuration alteration of surface morphology of the coke-based nitrogen-doped graphene sheets after activation is shown in Figure 9. It shows that the pristine GO had a stacked, multilayered structure. Subsequently, a wrinkled appearance was also found on the surfaces due to nitrogen doping. Several contributing mechanisms fueled the formation, such as restacking and coupling. Also, the folding of GO sheets was powered by the reduction effect of N-precursor ethylenediamine. The SEM images (Figure 9) also confirmed the rough texture of the surfaces with an increased heterogeneity of activated porous graphene oxide(a-PGO) and activated nitrogen-doped graphene oxide (A-NPGO). Heterogeneity at the surfaces is the predominant factor that enhances the surface area. This feature enhances the energy storage capacity of these ACs. They also contain a range of pores and cavities in various shapes and sizes. A summary of the performance of electrode materials used in supercapacitors is provided in Table 2.

**Table 2:** Summary of electrode performance on supercapacitor applications.

Electrode Materials	Carbon Source	Electrolyte	Specific Capacitance (F/g) @ Current Density (A/g)	Energy Density (Wh/kg)	Power Density (kW/kg)	Cyclic Stability	Capacitance Retention	Ref.
AC	Pet coke	TEABF_4_/AN	296 @ 1	20.2	32.8	2000	>91%	[101]
AC	Anthracite coal	6M KOH	280 @ 0.5	38.9	20	30,000	99%	[116]
AC	Pet coke	1M KOH	140 @ 0.5	20	250	1000	…	[3]
AC	Coal gasification fine ash	1 M Et_4_NBF_4_/AN	219.7 @ 1	13.4	0.475	15,000	82.6%	[117]
N, S-CLPACF	Coal liquefaction residue/polyacrylonitrile/terephthalic acid composite	6M KOH	267 @ 0.2	4.7	1.2	10,000	98%	[77]
AC	Semi coke	6M KOH	301 @ 1	…	…	10,000	95.9%	[102]
AC	High-ash coal liquefaction residue	6M KOH	250 @ 1	6.9	0.0625	10,000	99.7%	[78]
AC	Treated ultra-fine needle coke particles (UNCs)	6M KOH	387.2 @ 0.5	27.87	0.489	10,000	97.5%	[103]
Mn/N-APC	Pet coke	5M KOH	…	105	131.2Wh/kg	…	…	[118]
Nitrogen-doped porous carbon (CNPCs)	Anthracite coal	ethyl carbonate (EC)/diethyl carbonate (DEC)/dimethyl carbonate (DMC) was used as an electrolyte	56.1 @ 0.1	137.6	0.410	3000	97%	[80]
AC	Coal liquefaction residue	6M KOH	457 @ 0.5	46.5	10	10,000	93%	[81]
Porous carbon	Coal-based green needle coke	6M KOH	274.9 @ 1	20.51	1.0314	5000	95.6%	[82]
MoS_x_/ HPC-133	Refinery solid waste	6M KOH and 1 M Na_2_SO_4_	349.7 @ 1	33.99	0.450	10,000	…	[104]
Hierarchical porous carbon (HPC)	Coal tar pitch	6 M KOH	198 @ 1	6.45	0.483	4000	56.9%	[121]
AC	Anthracite coal	6 MKOH	243.6 @ 0.5	…	…	10,000	98.1%	[86]
AC	Low-quality subbituminous coal	1 M TEABF_4_ in Acetonitrile (ACN) 6 M KOH	7.00 @ 20 mV/s 57.63 @ 20 mV/s	1.40	13.775 25,920	1000	…	[84]
Hierarchical porous carbon	Hierarchical porous carbon	6.0 M KOH	268 @ 20	6.98	0.5	10,000	…	[88]
AC	Pitch coke and pet coke	ZnSO_4_ solution	146.4 mAh/g @ 0.1 A/g	117	15.8	5000	95%	[98]
PAC	Pet coke	3-M KOH	470 @ 0.5	16.3	0.062	15,000	98%	[97]
AC Graphitized carbon	Anthracite coal	mixed solvent of ethylene carbonate/ethyl methyl carbonate/dimethyl carbonate	302 @ 0.2 mV/s 568.1 mAh/g at 0.1 A/g	315.1	0.300	10,000 300 10,000	75% 97% 78%	[90]
Hierarchical porous carbon	Petroleum asphalt	6MKOH	93.3 @ 1	12.95	0.250	20,000	98%	[108]
Carbon composite	Asphaltene	1 M H_2_SO_4_	112 @ 0.4	8.6	0. 645	2000	-	[123]
Ni_3_S_2_/HPCs	Pet coke	1 M Na_2_SO_4_ solution	360 @ 1	19.65	0.450	20,000	86 %	[96]
Multi-heteroatom self-doped graphene	Pet coke	1 M H_2_SO_4_ electrolytes	44 @ 0.5	8.8	0.800	3000	98.78%	[95]
Porous graphene	Fuel coke	6 M KOH	167 @ 1	14	0.812	5000	90%	[110]
High-value capacitive carbon	Coal-coke	6 M KOH	191 @ 0.5	…	…	3000	92 %	[91]
Carbon nano-onions (CNOs)	Pet coke	6 M KOH	58 @ 1	7.47	0.221	10,000	94.4%	[94]
Sulfur-doped porous carbon composite	Pet coke	1 M Na_2_SO_4_	110.4 @ 0.5	49.7	0.450	5000	96.5%	[93]
AC	Pet coke	1 M LiPF_6_ in EC:DMC:DEC	145 @ 0.1	80	8.4	10,000	85%	[92]

### 3.3. Performance of Carbon Materials as Battery Electrodes

Many studies have proven the potential of electrodes for LIBs and potassium-ion and sodium-ion batteries when tested in standardized coin cells with the appropriate electrolyte. Non-nanoporous graphitic carbon was successfully fabricated using coal and used as an electrode material for LIBs. At a current density of 500 mA/g, the CG-2500 exhibited an initial discharge capacity of 165 mAh/g. At 174 mAh/g capacity after 1000 cycles, the material showed no capacitance deterioration with exceptional Coulombic efficiency (99.8%). This material may replace conventional graphite due to its low cost and strong cycle stability [111]. Coal was transformed into coal-based carbon (CBC) through catalytic activation and low-temperature graphitisation. The amorphous carbon in the coal was transformed into crystalline carbon using low-temperature graphitization. Following the raw coal’s catalytic graphitization, the CBC electrode demonstrated improved reversibility by delivering discharge and charge capacities of 531.3 and 222.7 mAh/g at a current density of 0.2 C and a Coulombic efficiency of 41.9%. After 100 cycles with a reversible 243 mAh/g capacity at a current density of 0.2 C, the CBC electrode demonstrated good long-term cycling stability with a Coulombic efficiency of over 98%. At 5C, it also showed a strong rate performance of 78.7 mAh/g [125].

The carbon nanosheets from coal tar pitch (CTP-CNSs) showed exceptional long-term cycling stability. At 2 A/g after 500 cycles, it had a Coulombic efficiency of 99.7% with a reversible 623 mAh/g capacity. It also exhibited a remarkable initial reversible capacity of 1147 mAh/g at 0.05 A/g and an exceptional rate capacity of 510 mAh/g at a high current density of 2 A/g [112]. N, S co-doped porous carbon-derived composite material was tested for battery electrode performance. It demonstrated excellent capability of capturing lithium polysulfides (LiPSs), which causes erosion and restricts the commercial production of LIBs. The initial discharge capacity of the PCPC/NiCoP/S was 1207.8 mAh/g, whereas the PCPC/S demonstrated 743.9 mAh/g at 0.2C. After 800 cycles, the PCPC/NiCoP-based electrode showed a notable discharge capacity (753.9 mAh/g) with a Coulombic efficiency of more than 98%. The PCPC matrix’s encapsulation of NiCoP nanoparticles improved both the adsorbability and conversion reaction kinetics of LiPSs [113]. Zhang et al. focused on the effect of using a green organic template in producing hierarchical porous carbon. Coal tar pitch was utilized as a raw material for this purpose, and the coral-like structure efficiently accelerated the contact area between the electrolyte and electrodes. The numerous defects and formed nanopores allowed high-flux and quick lithium storage. At a current density of 0.1A/g, the HPC-3 electrode showed an initial discharge capacity of 1066.8 mAh/g. It retained a high discharge capacity of 660 mAh/g after 1400 cycles at 1 A/g, and it reached an exceptional rate capacity of 219 mAh/g at 10 A/g [45]. 

Fluidized catalytic cracking (FCC) produced slurry oil (SO) to make needle coke and anode for LIBs. Hydrotreatment and vacuum distillation decreased the solid content and asphaltenes in the slurry oil to 0.0106 wt.% and below 0.1 wt.% because they are not advantageous for effective carbonization. A 15% yield was obtained by raising the distillation depth to 530 °C. Finally, a solvent extraction procedure was used to boost aromaticity to meet high-grade needle coke production specifications. The electrode materials produced with needle coke were assessed for their electrochemical performance. The crystalline structure of needle coke was revealed using XRD. Carbonaceous mesopore formation is crucial for carbonaceous material to be an effective electrode material for LIBs. This was accomplished in this work by using a lengthy reaction period at 420 °C for thermal polymerization and condensation of aromatic feedstocks. The greatest discharge capacity of the needle coke made from the extracted slurry oil (ESO-NC) was 379 mAh/g at 100 mA/g during the first circle. It showed an exceptional 99.7% Coulombic efficiency after 400 cycles, with a discharge capacity of 275 mAh/g and a current density of 500 mA/g [126]. To evaluate the applicability of waste carbon-based electrode materials as electrodes of both potassium and sodium-ion batteries, carbon materials (WFC) were produced from coal-based filter materials. A higher annealing temperature was reached during the process, which pushed the growth of a layered structure that was helpful for ion storage. WFC-1400 had a 224.7 mAh/g reversible capacity at 0.05 A/g initially. WFC-1400 demonstrated a discharge capacity of 245.6 mAh/g at 0.05 A/g and a capacitive retention of 93.7% after 50 cycles. It was shown that maintaining the nano graphite-like domain was essential for storing potassium and sodium ions. The layered structure of these nano graphite-like structures created many closed pores for Na^+^ to be filled in. They also provided a large number of active sites for K^+^ insertion. Here, the produced grain boundaries boosted the diffusion of ions [114].

Anthracite coal-derived porous carbon was prepared using NaCl as the template and NaOH as the activator at the initial stage. Subsequently, high-temperature annealing was applied. Following that, the modified Hummers technique was employed to produce graphene successfully. Because of its porous structure, coal-based porous carbon exhibited exceptional electrochemical activity when used as an anode for sodium-ion batteries without additional conductive agents. The initial discharge/charge capacity of coal-based graphene (rCG) at 50 mA/g was 1162/332 mAh/g. After the first ten activation cycles at a high current density of 500 mA/g, the rCG sample’s specific capacity was 198.2 mAh/g. After 100 cycles with 88.4% capacity retention, the specific capacity gradually decreased to 175.3 mAh/g. Remarkably, the specific capacity remained nearly unchanged after 1000 lengthy cycles. The graphene’s porous nature contributed to the coal-based carbon’s exceptionally prolonged cyclic capability. The insertion and release of sodium ions were facilitated by this porous assembly, which also successfully reduced the shift of volume that occurred during this process [127]. By pyrolyzing a combination of biomass waste (pomelo peel) and coal waste (bitumen), a hard-soft carbon (HC-SC) nanocomposite was created that was utilized as the anode material for sodium-ion batteries (SIBs). The HC-SC composite’s initial discharge/charge capacity was 261 and 242 mAh/g, which translates into a noticeably better initial Coulombic efficiency (ICE) of 92.7%. Because of its low BET-specific surface area, which lessened solid electrolyte interphase (SEI) generation, the HC-SC exhibited high ICE. After 100 cycles, the HC-SC composite still had its original porous structure, demonstrating its remarkable electrochemical stability. At 50 mA/g, the HC-SC was found to have a 241 mAh/g discharge capacity [128].

A convenient and environmentally friendly molten-salt process was used to create ultrathin N-doped porous carbon nanosheets (N-PCNS) from inexpensive coal. N-PCNS demonstrated exceptional performance as an electrode for LIBs. N-doping accelerated the accumulation of nanoflakes, which were ultrathin and graphene-like. After N doping, the resulting porous carbon nanosheets (PCNS) included ultrathin graphene-like nanoflakes that had aggregated. Also, it enhanced the holes developed due to the interaction between carbon nanosheets. Compared to PCNS (0.718 cm^3^/g), the N-PCNS exhibited a superior total pore volume (0.787 cm^3^/g). Rich ion transport channels in the N-PCNS led to outstanding electrochemical performance. N-PCNS, according to the electrochemical study, exhibited an initial Coulombic efficiency of 59.7% and an initial discharge/charge capacity of 923.8/518.4 mAh/g at a current density of 0.2 A/g. After 150 cycles with a Coulombic efficiency of almost 100%, it demonstrated a higher rate performance of 693.5 mAh/g at 0.1 A/g and produced a reversible capacity of 727.0 mAh/g at 0.2 A/g. Additionally, it showed a nearly 100% Coulombic efficiency and a discharge capacity of 175 mAh/g after 1000 cycles [129]. 

A vapor deposition technique created coal-based carbons covered with a pitch-based soft carbon layer (PCLC) for possible usage as sodium-ion battery (SIB) anodes. The volatile species obtained from pitch promoted an effective cross-linking reaction within hydroxyl-containing volatile species and oxygen-rich functional groups of coal. It formed a microcrystalline structure with a disordered inner phase and a dominant pseudo-graphitic phase in carbon derived from coal. The external layer of soft carbon coating boosted the electrical conductivity of coal-based carbon and significantly decreased surface imperfections. After 1000 cycles, the PCLC-1 electrode showed outstanding cycle stability, with 93.4% retention. At a current density of 0.3 A/g, PCLC-1’s initial reversible capacity was 246.8 mAh/g. In comparison to its pristine coal-based pyrolytic carbon, which had a capacity of 290.2 mAh/g and an initial Coulombic efficiency (ICE) of 59.9%, the PCLC-1 electrode produced a better Na-storage capacity of 312.2 mAh/g with a high ICE of 85.3%. Furthermore, at 0.5 A/g, the PCLC-1 electrode demonstrated remarkable rate performance, achieving 188.2 mAh/g [130].

**Figure 10:**
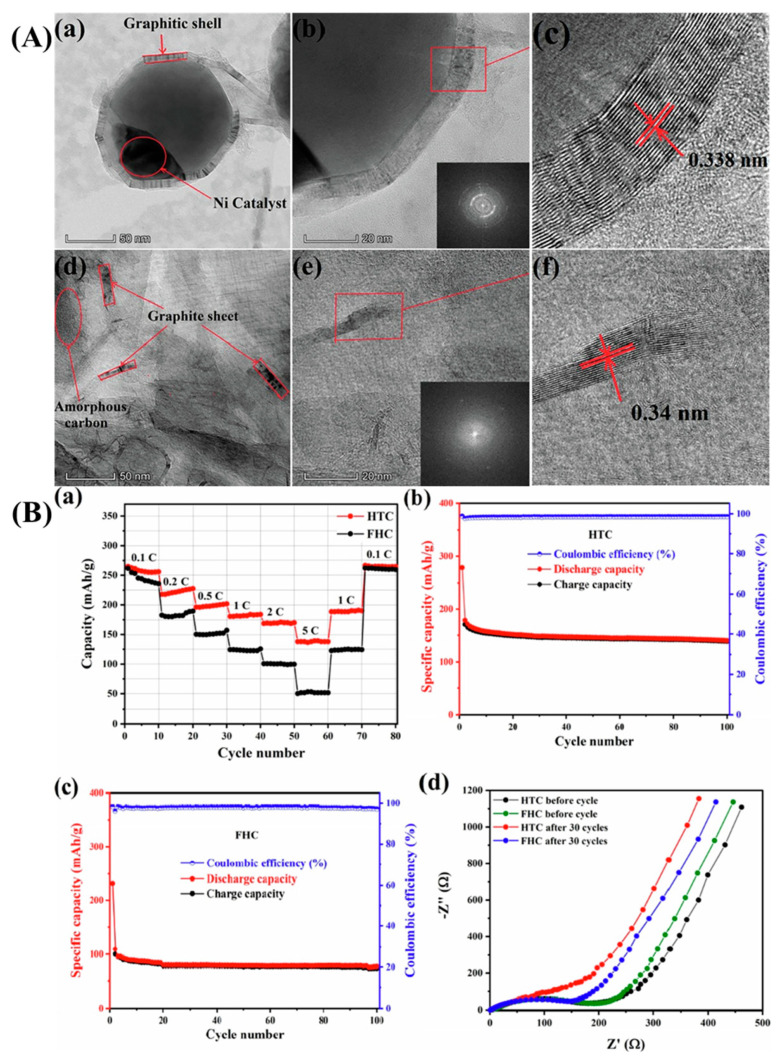
(**A**) TEM images of nickel-treated graphite (FHC-Ni) by flash joule heating process (FJH) and graphite produced without applying nickel catalysts in the FJH. (**B**) Performance analysis of HTC and FHC electrodes: (**a**) comparison of rate performance, (**b**,**c**) long-term cycling stability at a 2C current rate, (**d**) electrochemical impedance spectroscopy (EIS) profiles pre- and post-30 charge–discharge cycles. Reproduced with permission [120]. Copyright 2023, Elsevier.

Coal-based mesocarbon microbeads (CMCMB) were prepared using co-carbonization, and their potential as an electrode material for LIBs was tested [131]. At 100 mA/g, the initial discharge and charge capacities were 773.7/472.3 mAh/g (CMCMB-3) and 833.1/486.2 mAh/g (CMCMB-5). Following 400 cycles, CMCMB-3’s reversible capacity was 394.5 mAh/g with 99.6% Coulombic efficiency at a current density of 500 mA/g. With a current density of 0.1 A/g, CMCMB-3 demonstrated an ultra-high-rate capacity of 426.5 mAh/g. The CMCMB-3’s reversible capacity was 451.5 mAh/g following 50 cycles of charging and discharging. While CMCMB-5 had greater initial discharge/charge capacity (833.1/486.2 mAh/g) than CMCMB-3, CMCMB-5’s rate capability and long-term cyclic efficiency were somewhat worse. CMCMB-5 had a high carbon black coating and a low π-π connected organized architecture, which decreased Li^+^ insertion capacity and electrical conductivity [132,133]. Two techniques were used for the catalytic graphitization of coal: flash Joule heating (FJH) and high-temperature heating (HTC). They showed how coal was successfully structurally converted from an amorphous to a crystalline form using Ni catalysts. A common circular nanostructure was observed in the Ni-diffused graphitic carbon (FHC-Ni). Figure 10A depicts the presence of dark spots in a vast area. The dark points resembled the nickel particles embedded in carbon-rich graphite. During the graphitization process in FHC, when catalysts were moved over the carbon, they formed a circular ring-like structure with various curvatures, illustrated in Figure 10A. The crystalline ordering of the graphitic carbon was also observed in Figure 10A. In the subsequent stages, an acid wash and wash with deionized water nearly removed all the nickel particles from the graphite. Therefore, nickel particles had been seen in the samples. The amorphous carbon containing layered graphitic sheets is also seen in Figure 10A, which indicates a strong crystal structure. However, the inter-layer distance was slightly altered when acid washing was applied. When utilized as an anode, these graphitized carbons demonstrated exceptional cycle stability and rate capability. Figure 10B showcases a comprehensive evaluation of the electrochemical behaviors of HTC and FHC electrodes, showing their rate capabilities and how each responds to different current demands. Also, an analysis of their cycling performances at a 2C rate revealed the endurance and capacity retention over time. Lastly, it compared the EIS curves of both electrodes before and after undergoing 30 cycles, providing insight into their resistance changes and ion transport dynamics with usage. The carbon derived from the flash-heated method (FHC) in LIBs exhibited an initial Coulombic efficiency of 71.3% and an initial charge/discharge capacity of 321.3/450.6 mAh/g at 0.1C. Following 100 cycles, the capacity was 166.1 mAh/g, and at a 2C current rate, the initial Coulombic efficiency (ICE) was close to 100%. On the other hand, FHC’s charge/discharge capacity in potassium ion batteries (PIBs) was 262.2/498.1 mAh/g at a current density of 0.1C and an ICE of 52.6%. In this instance, it retained 97% of its capacity after 100 cycles at a current density of 2C, maintaining a capacity of 73.88 mAh/g. In both LIBs and PIBs, the HTC anode outperformed the FHC anode electrochemically by a small margin. Despite these outcomes, a furnace is used at a high temperature to complete the traditional heating process, which requires significant time and energy. The FJH heating method, on the other hand, was straightforward. It produced high temperatures within a very short time and provided ultrafast cooling. This suggests that compared to the existing conventional heating method, the FJH is far more effective considering the time and energy needs. Consequently, producing anode materials for LIBs and PIBs using the catalytic synthesis process based on FJH may prove to be a cost-effective and sustainable substitute, while coal-based carbons may prove to be a viable substitute for natural graphite [120]. 

Green mesocarbon microbeads (GMCMBs) were carbonized at 1000 °C to create carbonized mesocarbon microbeads (CMCMBs). Before that, mesophase pitch (MP) from FCC decant oil was emulsified with different anisotropic components to create GMCMBs. The anisotropic content of the associated mesophase pitch rose from 12.3 vol% to 98.3 vol%, and the stacking order degree of aromatic molecules of GMCMBs steadily increased along with the condensation degree. With a specific surface area and total pore volume of 390.35 m^2^/g and 0.212 cm^3^/g, respectively, CMCMB-12 exhibited the greatest values. At 100 mA/g, it demonstrated an initial charge/discharge capacity of 782.5/473.0 mAh/g. When the current density was 100 mA/g, CMCMB-12 provided 437 mAh/g. With a modest mesopore volume (0.029 cm^3^/g) and a large micropore volume (0.183 cm^3^/g), CMCMB-12’s extensive pore structure was primarily responsible for its better rate capacity. A low current density of 100 mA/g was achieved with the reversible capabilities of CMCMB-12 after 200 cycles of charging and discharging [131]. Various studies have demonstrated the efficacy of electrodes for lithium-ion, potassium-ion, and sodium-ion batteries, validated through tests in standard coin cells with suitable electrolytes, as summarized in Table 3.

Carbon materials’ long-term stability and reusability are promising aspects of energy storage. However, other strategic efforts to examine other factors like processing conditions, structural integrity, resistance to degradation, and the ability to retain electrochemical properties are critical in promoting stability. Separating these characteristics through specific treatments and engineered carbon structures offers durability and lifetime guarantees of such materials in numerous charge–discharge events. Studies have extensively researched the reusability and stability of carbon materials for energy storage applications. Carbon materials in porous carbon, nanowires, and nanofibers have been highly considered for electrochemical energy storage due to their good chemical stability, high electronic conductivity, and large specific surface area [134,135,136]. This has led to using carbon materials for supercapacitors, lithium-ion, and sodium-ion batteries [28,137,138]. Notably, heteroatom doping and manipulation of the dimensionality of carbon materials have achieved significantly high energy density in supercapacitors [139]. The interconnected porous structure in carbon materials also considerably enhances energy storage by aiding in electrolyte storage and simultaneous electron transfer. Furthermore, as carbon fibers derived from biomass possess these properties, the reusability of carbon-based materials is well known in energy storage. Additionally, the widely known stability and surface chemistry of carbon-based materials make them a viable option for various catalytic reactions. The other major challenge of carbon-based electrode materials is the low energy density, low potential for further energy density, and the recycling potential of this material to obtain their economic and environmental advantage over the other. Considering the ecological importance of sustainability and recycling of materials, carbon-based electrode materials for supercapacitors have improved. The research on carbon materials in the literature can support stability and reusability, validating these materials as viable alternatives for electrical energy storage devices. The unique material properties of carbon materials, combined with the optimization of these materials in different energy storage devices, promote recycling reusability for sustainable electrical energy generation [140,141,142].

**Table 3:** Summary of electrode performance on battery applications.

Materials	Carbon Precursor	Battery Type	Electrolyte	Initial Discharge Capacity (mAh/g)	Final Discharge (mAh/g)	No. of Cycles	Coulombic Efficiency (%)	Ref.
CG-2500	Coal	Lithium-ion battery	1 M LiPF_6_ in EC/PE	165 @ 0.5 A/g	174 @ 0.5 A/g	1000	99.8%	[111]
FJH	Coal	Lithium-ion battery Potassium-ion battery	1 M LiPF_6_ in EC/DEC 0.8 M KPF_6_ in EC/DEC	450.6 at (0.1C) 498.1 at (0.1C)	166.1 at (2C) 73.88 at (2C)	100 100	99% 97%	[120]
PCPC/NiCoP	Pet coke	Lithium–sulfur battery	DOL/DME/LITFSI	1207.8 mAh/g at 0.2C	753mAh at 2C	800	>98%	[113]
HPC-3	Coal tar pitch (CTP)	Lithium-ion battery	1M LiPF_6_/EC/DC/EMC	1066.8mAh/g at 0.1 A/g	660 @ 1 A/g	1400	…	[45]
CTP-CNSs	Coal tar pitch	Lithium-ion battery	1 M LiPF_6_ EC/DEC	1322 @ 0.05 A/g	623 @ 2A/g	500	99.7%	[112]
ESO-NC	Slurry oil (SO) from fluidized catalytic cracking (FCC)	Lithium-ion battery	…	379 @ 100 mA/g	275 @ 500 mA/g	400	99.7%	[126]
LiMn_2_O_4_/bituminous coal composite	Bituminous coal	Lithium-ion battery	1M LiSO_4_ DI water	…	…	…	…	[82]
WFC-1400 WFC-1800	Waste coal	Potassium-ion batteries (PIBs) Sodium-ion batteries (SIBs)	0.8 M KPF_6_ in EC/DEC 1M NaPF_6_ in DME	224.7 mAh/g at 0.05 A/g 241.7 mAh/g at 0.02 A/g	245.6 mAh/g at 0.05 A/g 239.0 mAh/g at 0.02 A/g	50	93.7% 102.5%	[114]
Coal-based graphene (rCG)	Coal	Sodium-ion batteries	0.8 M NaClO_4_ in EC/DEC	1162 mA h/g at 50 mA/g.	175.3 mAh/g at 500 mA/g	1000	88.4%	[127]
Hard–soft carbon (HC-SC)	Biomass (pomelo peel) and coal waste (bitumen)	Sodium-ion batteries	…	261 mAh/g at 50 mA/g	241 mAh/g at 50 mA/g	100	92.3%	[128]
N-PCNS	Coal	Lithium-ion battery	…	923.8 mAh/g at 0.2 A/g	175 mAh/g at 5 A/g	1000	100%	[129]
PCLC-1	Coal	Sodium-ion batteries	1 M NaClO_4_ in EC/DEC/FEC	246.8 mAh/g at 0.3 A/g	230.5 mAh/g at 0.3 A/g	1000	93.4%	[130]
Coal-based carbon (CBC)	Coal	LIBs	1.0M LiPF_6_ in EC/DEC	531.3 mAh/g at 0.2C	243 mAh/g at 0.2C	100	98%	[125]
MCMB-2	Coal tar	LIBs	1 M LiPF_6_ in EC/DMC/DEC	639 mAh/g at 0.04C	336 mAh/g at 0.04C	60	…	[143]
CMCMB-3	FCC and CCB	LIBs	1 M LiPF_6_ in EC/DMC	773.7 mAh/g at 100 mA/g.	394.5 mAh/g at 500 mA/g	400	99.6%	[115]
P-HC	coal	sodium-ion batteries	1.0 M NaClO_4_ in PC/EC/FEC	284.4 mAh/g at 20 mA/g	212.1 mAh/g at 100 mA/g	500	…	[144]
MoO_2_@coal gangue	coal gangue	Li–O_2_ batteries	…	9748 mAh/g at 100 mA/g	…	…	…	[145]
CMCMB-12	FCC decant oi	LIBs	1.0 M LiPF_6_ in EC/DEC	782.5 mAh/g at 100 mA/g	405 mAh/g at 500 mA/g	200	100%	[131]

## 4. Economic Aspects of Carbon-Based Energy Storage Materials

LIBs are the dominant technologies for electric vehicles and large-scale grid battery storage. Projections from the International Energy Agency (IEA) suggest a substantial rise in LIB demand, shooting up from 1.57 TWh in 2022 to 6.79 TWh by 2030 [146]. While various cathodes are utilized in current LIB cells depending on power requirements, nearly all employ graphite anodes. This trend indicates a steady rise in graphite demand for LIBs, expected to reach almost 3.5 Mt/y by 2040, compared to just 0.14 Mt/y in 2020 [147]. Graphite sources include natural and synthetic varieties derived from low-value refineries and coal by-products. Synthetic graphite is increasingly used for battery anode materials due to its superior electrochemical performance and high purity. Due to the rapid increase in graphite demand for anode production, the cost of graphite is also rising. Depending on the grade of graphite, the cost varies from USD 1000 to USD 20,000 per ton of graphite [148,149]. 

The escalating demand for LIBs strains raw material supplies, leading to a surge in lithium prices [150]. Consequently, sodium emerges as a viable and sustainable alternative, given its abundance and lower cost. SIBs are beginning commercial production and are poised to gain traction for stationary storage and small vehicle applications [151]. However, conventional graphite is not suitable for SIB anodes due to intercalation issues and unstable Na-C compounds [152]. Instead, hard carbon arises as a promising alternative, synthesized from various feedstocks, predominantly from petroleum pitch and coal tar [153]. Forecasts suggest that SIBs could boost demand for hard carbon to approximately 0.7 Mt/y by 2030, with anticipated prices ranging from USD 8000 to USD 30,000 per ton, based on laboratory-scale predictions, as commercial data are not yet available [154,155]. However, the advent of mass production is expected to reduce costs significantly. Beyond these electrode materials, porous activated carbon finds applications in supercapacitors and energy storage. In 2022, the market value of activated carbon used in supercapacitors stood at USD 116 million, with projections indicating that it will surpass USD 357 million by 2031 [156]. The market price for superior-grade activated carbon for supercapacitor applications ranges from USD 15,000 to USD 50,000 per ton [157]. Nonetheless, pricing is heavily influenced by the type of feedstock materials and the specific grade of activated carbon required for applications. 

Furthermore, the growing demand for energy storage solutions underscores the importance of innovative materials and technologies. As the world transitions to renewable energy sources and electrification, there is growing promise in utilizing value-added energy storage materials derived from low-value petroleum by-products. This approach contributes to achieving net-zero targets of the petrochemical industries and presents a sustainable energy storage solution.

## 5. Conclusions and Prospects

Pursuing sustainable and efficient energy storage solutions stands as a cornerstone in the global transition towards renewable energy sources and the reduction of carbon footprints. This review paper delves into the innovative approach of repurposing waste materials from oil refineries and coal-processing plants as precursors for carbon-based electrodes. This strategy bridges the gap between advances in energy storage and environmental sustainability by transforming waste into high-performance electrode materials. This approach addresses waste disposal challenges and heralds the development of next-generation energy storage systems. Investigating waste-derived carbon materials for energy storage signifies a pivotal advancement towards sustainable and efficient technologies. 

Our analysis reveals that several waste materials like semi-coke, coal gasification fine ash, coal tar pitch, pet coke, and petroleum vacuum residue exhibit substantial potential as carbon precursors for electrode fabrication. Enriched with high-carbon content and easily moldable through processes like carbonization and activation, these materials are strong candidates for electrodes that meet the rigorous demands of modern batteries and supercapacitors. Converting these industrial by-products into valuable electrode materials emphasizes the need to optimize the processing conditions. Techniques like carbonization and activation with KOH play a crucial role in enhancing the electrochemical attributes of carbon materials. 

Furthermore, heteroatom doping significantly enhances surface interactions, facilitates ion diffusion, and boosts storage capacities. This review sheds light on the electrochemical performance of waste-derived carbon electrodes, illustrating their competence across various energy storage devices. When juxtaposed with traditional electrode materials, waste-derived carbons demonstrate encouraging outcomes, suggesting their potential to replace or augment existing materials in energy storage solutions. 

The journey towards fully leveraging the capabilities of waste-derived carbon materials for energy storage is ongoing and laden with potential areas for further exploration and innovation. Future research endeavors should deepen the understanding of the interplay between the microstructural characteristics of waste-derived carbons, such as porosity and heteroatom content, and their electrochemical behavior. Employing advanced characterization techniques could unlock new insights, guiding the optimization of carbon materials for superior performance. 

The exploration of novel fabrication techniques capable of precise control over the structure and composition of carbon materials is crucial. Techniques like templating, electrospinning, and 3D printing are promising avenues for creating electrodes with customized microstructures and functionalities. Despite recognizing the advantages of heteroatom doping, systematic studies to ascertain the most beneficial doping elements and concentrations for maximizing electrode performance are essential. 

Translating laboratory-scale discoveries into real-world applications poses a significant challenge. Efforts should concentrate on integrating waste-derived carbon electrodes into commercial battery and supercapacitor systems, evaluating their performance and cost-effectiveness at a larger scale. Addressing manufacturing challenges and ensuring material consistency is critical for commercialization.

Assessing the environmental impact of producing and using waste-derived carbon materials is vital beyond their electrochemical performance. Life cycle assessments can offer insights into the sustainability of these materials, aiding in the development of genuine green energy storage solutions. Adopting waste-derived carbon materials in energy storage could benefit from supportive regulatory and policy frameworks, encouraging industrial waste recycling into valuable products and setting standards for material performance and safety. 

Tackling the multifaceted challenges related to waste-derived carbon materials for energy storage necessitates interdisciplinary collaboration. Uniting experts from diverse fields can catalyze holistic development, ranging from fundamental research to application. Waste-derived carbon materials present a promising path for propelling energy storage technologies forward while addressing environmental sustainability. These advancements set a foundation for further research and development aimed at unlocking the full potential of these materials. Significant strides can be made toward a sustainable energy future by exploring innovative fabrication methods, optimizing material properties, and integrating these materials into practical energy storage solutions. The path ahead is complex and challenging but is ripe with the promise of transforming waste into valuable resources in the realm of energy storage.

## Data Availability

Data are contained within the article.

## References

[1] Skyllas-Kazacos M. (2010). Electro-Chemical Energy Storage Technologies for Wind Energy Systems. Stand-Alone and Hybrid Wind Energy Systems: Technology, Energy Storage and Applications.

[2] Solaun K., Cerdá E. (2019). Climate Change Impacts on Renewable Energy Generation. A Review of Quantitative Projections. Renew. Sustain. Energy Rev..

[3] Shah S.S., Aziz M.A., Yamani Z.H. (2023). High-Yield Petroleum Coke Derived Activated Carbon with Self-Sulfur Confinement for High-Performance Supercapacitor. Diam. Relat. Mater..

[4] Shah S.S., Niaz F., Ehsan M.A., Das H.T., Younas M., Khan A.S., Rahman H.U., Nayem S.M.A., Oyama M., Aziz M.A. (2024). Advanced Strategies in Electrode Engineering and Nanomaterial Modifications for Supercapacitor Performance Enhancement: A Comprehensive Review. J. Energy Storage.

[5] Shah S.S., Aziz M.A., Rasool P.I., Mohmand N.Z.K., Khan A.J., Ullah H., Feng X., Oyama M. (2024). Electrochemical Synergy and Future Prospects: Advancements and Challenges in MXene and MOFs Composites for Hybrid Supercapacitors. Sustain. Mater. Technol..

[6] Shah S.S., Aziz M.A. (2024). Properties of Electrode Materials and Electrolytes in Supercapacitor Technology. J. Chem. Environ..

[7] Fan L.-Q., Liu G.-J., Zhao J.-C., Wu J.-H., Zhong J., Lin J.-M., Huo J.-H., Liu L. (2014). Facile One-Step Hydrothermal Syntheses and Supercapacitive Performances of Reduced Graphene Oxide/MnO_2_ Composites. Compos. Sci. Technol..

[8] Lopes P.P., Stamenkovic V.R. (2020). Past, Present, and Future of Lead–Acid Batteries. Science.

[9] Dhundhara S., Verma Y.P., Williams A. (2018). Techno-Economic Analysis of the Lithium-Ion and Lead-Acid Battery in Microgrid Systems. Energy Convers. Manag..

[10] Shah S.S., Qasem M.A.A., Berni R., Del Casino C., Cai G., Contal S., Ahmad I., Siddiqui K.S., Gatti E., Predieri S. (2021). Physico-Chemical Properties and Toxicological Effects on Plant and Algal Models of Carbon Nanosheets from a Nettle Fibre Clone. Sci. Rep..

[11] Titirici M.-M., White R.J., Brun N., Budarin V.L., Su D.S., del Monte F., Clark J.H., MacLachlan M.J. (2015). Sustainable Carbon Materials. Chem. Soc. Rev..

[12] Mauter M.S., Elimelech M. (2008). Environmental Applications of Carbon-Based Nanomaterials. Environ. Sci. Technol..

[13] Hagemann N., Spokas K., Schmidt H.-P., Kägi R., Böhler M., Bucheli T. (2018). Activated Carbon, Biochar and Charcoal: Linkages and Synergies across Pyrogenic Carbon’s ABCs. Water.

[14] Xu C., Strømme M. (2019). Sustainable Porous Carbon Materials Derived from Wood-Based Biopolymers for CO2 Capture. Nanomaterials.

[15] Shah S.S., Aziz M.A., Ali M., Hakeem A.S., Yamani Z.H. (2023). Advanced High-Energy All-Solid-State Hybrid Supercapacitor with Nickel-Cobalt-Layered Double Hydroxide Nanoflowers Supported on Jute Stick-Derived Activated Carbon Nanosheets. Small.

[16] Aziz M.A., Shah S.S., Mahnashi Y.A., Mahfoz W., Alzahrani A.S., Hakeem A.S., Shaikh M.N. (2023). A High-Energy Asymmetric Supercapacitor Based on Tomato-Leaf-Derived Hierarchical Porous Activated Carbon and Electrochemically Deposited Polyaniline Electrodes for Battery-Free Heart-Pulse-Rate Monitoring. Small.

[17] Ghanem A.S., Ba-Shammakh M., Usman M., Khan M.F., Dafallah H., Habib M.A.M., Al-Maythalony B.A. (2020). High Gas Permselectivity in ZIF-302/Polyimide Self-consistent Mixed-matrix Membrane. J. Appl. Polym. Sci..

[18] Liu K., Ran Q., Li F., Shaheen S.M., Wang H., Rinklebe J., Liu C., Fang L. (2022). Carbon-Based Strategy Enables Sustainable Remediation of Paddy Soils in Harmony with Carbon Neutrality. Carbon Res..

[19] Masarapu C., Wang L., Li X., Wei B. (2012). Tailoring Electrode/Electrolyte Interfacial Properties in Flexible Supercapacitors by Applying Pressure. Adv. Energy Mater..

[20] Ania C.O., Pernak J., Stefaniak F., Raymundo-Piñero E., Béguin F. (2009). Polarization-Induced Distortion of Ions in the Pores of Carbon Electrodes for Electrochemical Capacitors. Carbon N. Y..

[21] Wang W., Cheng F., Zhang W., Li X. (2023). Biochar Made into Efficient Electrodes for Capacitive Deionization. J. Phys. Conf. Ser..

[22] Gang-Wei S., Wen-Hua S., Xiao-Jun L., Wen-Ming Q., Li-Cheng L. (2011). Asymmetric Capacitance Behavior Based on the Relationship between Ion Dimension and Pore Size. Acta Physico Chimica Sin..

[23] Dai Z., Ren P., Chen Z., Guo Z., Hou X., He W., Jin Y., Ren F. (2021). Pore- and Heteroatom-Controlled Superabsorbent-Resin-Derived Carbon Aerogels for Supercapacitors via Adjusting the Methylene Blue Concentration. Adv. Mater. Interfaces.

[24] Zhao C., Yang Z., Zhou X., Hao Z., Chen J., Wang Z., Chen X., Wu X., Li L., Li L. (2024). Recent Progress on Electrolyte Boosting Initial Coulombic Efficiency in Lithium-Ion Batteries. Adv. Funct. Mater..

[25] Kurc B. (2018). Sulfolane with LiPF6, LiNTf2 and LiBOB-as a Non-Flammable Electrolyte Working in a Lithium-Ion Batteries with a LiNiO2 Cathode. Int. J. Electrochem. Sci..

[26] Kang Y., Wang J., Du L., Liu Z., Zou X., Tang X., Cao Z., Wang C., Xiong D., Shi Q. (2019). Overcharge Investigations of LiCoO 2 /Graphite Lithium Ion Batteries with Different Electrolytes. ACS Appl. Energy Mater..

[27] Winter M., Brodd R.J. (2004). What Are Batteries, Fuel Cells, and Supercapacitors?. Chem. Rev..

[28] Shah S.S., Aziz M.A., Al Marzooqi M., Khan A.Z., Yamani Z.H. (2024). Enhanced Light-Responsive Supercapacitor Utilizing BiVO4 and Date Leaves-Derived Carbon: A Leap towards Sustainable Energy Harvesting and Storage. J. Power Sources.

[29] Shaheen Shah S., Abu Nayem S.M., Sultana N., Saleh Ahammad A.J., Abdul Aziz M. (2022). Preparation of Sulfur-Doped Carbon for Supercapacitor Applications: A Review. ChemSusChem.

[30] Shah S.S., Aziz M.A., Cevik E., Ali M., Gunday S.T., Bozkurt A., Yamani Z.H. (2022). Sulfur Nano-Confinement in Hierarchically Porous Jute Derived Activated Carbon towards High-Performance Supercapacitor: Experimental and Theoretical Insights. J. Energy Storage.

[31] Haque M.A., Akanda M.R., Hossain D., Haque M.A., Buliyaminu I.A., Basha S.I., Oyama M., Aziz M.A. (2020). Preparation and Characterization of Bhant Leaves-Derived Nitrogen-Doped Carbon and Its Use as an Electrocatalyst for Detecting Ketoconazole. Electroanalysis.

[32] Shah S.S., Aziz M.A., Yamani Z.H. (2022). Recent Progress in Carbonaceous and Redox-Active Nanoarchitectures for Hybrid Supercapacitors: Performance Evaluation, Challenges, and Future Prospects. Chem. Rec..

[33] Aziz M.A., Shah S.S., Aziz M.A., Shah S.S. (2023). Biomass-Based Supercapacitors.

[34] Kothandam G., Singh G., Guan X., Lee J.M., Ramadass K., Joseph S., Benzigar M., Karakoti A., Yi J., Kumar P. (2023). Recent Advances in Carbon-Based Electrodes for Energy Storage and Conversion. Adv. Sci..

[35] Chaudhary P., Bansal S., Sharma B.B., Saini S., Joshi A. (2024). Waste Biomass-Derived Activated Carbons for Various Energy Storage Device Applications: A Review. J. Energy Storage.

[36] Hong H., Tu H., Jiang L., Du Y., Wong C. (2024). Advances in Fabric-Based Supercapacitors and Batteries: Harnessing Textiles for next-Generation Energy Storage. J. Energy Storage.

[37] Zhang C., Jiang J., Guan Z., Zhang Y., Li Y., Song B., Shao W., Zhen L. (2024). Unveiling the Sp^2^─sp^3^ C─C Polar Bond Induced Electromagnetic Responding Behaviors by a 2D N-doped Carbon Nanosheet Absorber. Adv. Sci..

[38] Rice P.S., Lee G., Schwartz B., Autrey T., Ginovska B. (2024). Leveraging Curvature on N-Doped Carbon Materials for Hydrogen Storage. Small.

[39] Mei H., Mei X., He X., Bie Z., Cui J. (2024). In-Situ Investigation on Structure Transformation of Single-Walled Carbon Nanotubes. Appl. Surf. Sci..

[40] Lu S., Jin M., Zhang Y., Niu Y., Gao J., Li C.M. (2018). Chemically Exfoliating Biomass into a Graphene-like Porous Active Carbon with Rational Pore Structure, Good Conductivity, and Large Surface Area for High-Performance Supercapacitors. Adv. Energy Mater..

[41] Shah S.S., Aziz M.A., Usman M., Hakeem A.S., Ali S., Alzahrani A.S. (2023). Biomass-based Supercapacitors: Lab to Industry. Biomass-Based Supercapacitors.

[42] Basirun W.J., Saeed I.M., Saidur M.R., Ahmed S. (2022). Manganese Oxides-Graphene Nanocomposites as Advanced Supercapacitors. Encyclopedia of Energy Storage: Volume 1–4.

[43] Sharma P., Kumar V. (2019). Study of Electrode and Electrolyte Material of Supercapacitor. Mater. Today Proc..

[44] Şahin M.E., Blaabjerg F., Sangwongwanich A. (2022). A Comprehensive Review on Supercapacitor Applications and Developments. Energies.

[45] Zhang M., Qu M., Yuan W., Mu J., He Z., Wu M. (2023). Green Synthesis of Hierarchically Porous Carbon Derived from Coal Tar Pitch for Enhanced Lithium Storage. Batteries.

[46] Gao Z., Zhang Y., Song N., Li X. (2017). Biomass-Derived Renewable Carbon Materials for Electrochemical Energy Storage. Mater. Res. Lett..

[47] Huang Y., Tang Z., Zhou S., Wang H., Tang Y., Sun D., Wang H. (2022). Renewable Waste Biomass-Derived Carbon Materials for Energy Storage. J. Phys. D Appl. Phys..

[48] Zhu J., Roscow J., Chandrasekaran S., Deng L., Zhang P., He T., Wang K., Huang L. (2020). Biomass-Derived Carbons for Sodium-Ion Batteries and Sodium-Ion Capacitors. ChemSusChem.

[49] Liu M., Zhang Y., Xu Z., Han X., Gou W., Sun Y., Ming Li C. (2023). Highly Microporous Carbon from Enteromorpha for High-Performance Aqueous Zinc-chalcogen (S, SeS_2_) Batteries. Batter. Supercaps.

[50] Xu Z., Zhang Y., Gou W., Liu M., Sun Y., Han X., Sun W., Li C. (2022). The Key Role of Concentrated Zn(OTF)_2_ Electrolyte in the Performance of Aqueous Zn–S Batteries. Chem. Commun..

[51] Gul E., Shah S.A., Shah S.N.A. (2023). Chloride Salt-activated Carbon for Supercapacitors. Biomass-Based Supercapacitors.

[52] Elkasabi Y., Mullen C.A. (2021). Progress on Biobased Industrial Carbons as Thermochemical Biorefinery Coproducts. Energy Fuels.

[53] Dutta V., Verma R., Gopalkrishnan C., Yuan M.-H., Batoo K.M., Jayavel R., Chauhan A., Lin K.-Y.A., Balasubramani R., Ghotekar S. (2022). Bio-Inspired Synthesis of Carbon-Based Nanomaterials and Their Potential Environmental Applications: A State-of-the-Art Review. Inorganics.

[54] Yin Y., Liu Q., Zhao Y., Chen T., Wang J., Gui L., Lu C. (2023). Recent Progress and Future Directions of Biomass-Derived Hierarchical Porous Carbon: Designing, Preparation, and Supercapacitor Applications. Energy Fuels.

[55] Antal M.J., Allen S.G., Dai X., Shimizu B., Tam M.S., Grønli M. (2000). Attainment of the Theoretical Yield of Carbon from Biomass. Ind. Eng. Chem. Res..

[56] Islam T., Hasan M.M., Shah S.S., Karim M.R., Al-Mubaddel F.S., Zahir M.H., Dar M.A., Hossain M.D., Aziz M.A., Ahammad A.J.S. (2020). High Yield Activated Porous Coal Carbon Nanosheets from Boropukuria Coal Mine as Supercapacitor Material: Investigation of the Charge Storing Mechanism at the Interfacial Region. J. Energy Storage.

[57] (2020). Marine Benchmark Maritime CO_2_ Emissions. Res. Brief.

[58] Wang M., Wang Q., Li T., Kong J., Shen Y., Chang L., Xie W., Bao W. (2021). Catalytic Upgrading of Coal Pyrolysis Volatiles by Porous Carbon Materials Derived from the Blend of Biochar and Coal. ACS Omega.

[59] Manera C., Fragoso H.P., Agra A.A., Flores B.D., Osório E., Godinho M., Vilela A.C.F. (2023). Systematic Mapping of Studies on Coal Tar and Pitch over the Last Five Decades (1970–2023). Chem. Eng. Res. Des..

[60] Mahler B.J., Van Metre P.C., Crane J.L., Watts A.W., Scoggins M., Williams E.S. (2012). Coal-Tar-Based Pavement Sealcoat and PAHs: Implications for the Environment, Human Health, and Stormwater Management. Environ. Sci. Technol..

[61] Hu H., Wu M. (2020). Heavy Oil-Derived Carbon for Energy Storage Applications. J. Mater. Chem. A.

[62] Zhang H., He X., Gu J., Xie Y., Shui H., Zhang X., Xiao N., Qiu J. (2018). Wrinkled Porous Carbon Nanosheets from Methylnaphthalene Oil for High-Performance Supercapacitors. Fuel Process. Technol..

[63] Wei F., Bi H., Jiao S., He X. (2020). Interconnected Graphene-like Nanosheets for Supercapacitors. Acta Physico-Chimica Sin..

[64] Mo B., Wang L., Li L., Han B., Xu G., Wang K., Zhang J., Ni Z., He X., Zhou W. (2023). Supercapacitor Performances of Coal Tar Pitch Based Spherical Active Carbons Fabricated by the SiO_2_ Template Method. J. Chem. Eng. Japan.

[65] Gao F., Zang Y.H., Wang Y., Guan C.Q., Qu J.Y., Wu M.B. (2021). A Review of the Synthesis of Carbon Materials for Energy Storage from Biomass and Coal/Heavy Oil Waste. Xinxing Tan Cailiao/New Carbon Mater..

[66] Azizian S., Khosravi M. (2019). Advanced Oil Spill Decontamination Techniques. Interface Science and Technology.

[67] Boateng A.A. (2016). Rotary Kiln Petroleum Coke Calcination Process. Rotary Kilns.

[68] Boateng A.A. (2016). Combustion and Flame. Rotary Kilns.

[69] Okeke I.J., Adams T.A. (2019). Life Cycle Assessment of Petroleum Coke Gasification to Fischer-Tropsch Diesel. Computer Aided Chemical Engineering.

[70] Phillips R.D. (2005). Coal Tar. Encyclopedia of Toxicology.

[71] Ozbayoglu G. (2018). Energy Production From Coal. Compr. Energy Syst..

[72] Roberts L. (2014). Coal Tar. Encyclopedia of Toxicology.

[73] Basha S.I., Aziz A., Maslehuddin M., Ahmad S., Hakeem A.S., Rahman M.M. (2020). Characterization, Processing, and Application of Heavy Fuel Oil Ash, an Industrial Waste Material—A Review. Chem. Rec..

[74] Huth M., Heilos A. (2013). Fuel Flexibility in Gas Turbine Systems: Impact on Burner Design and Performance. Modern Gas Turbine Systems: High Efficiency, Low Emission, Fuel Flexible Power Generation.

[75] Rauf M., Shah S.S., Shah S.K., Shah S.N.A., Haq T.U., Shah J., Ullah A., Ahmad T., Khan Y., Aziz M.A. (2022). Facile Hydrothermal Synthesis of Zinc Sulfide Nanowires for High-Performance Asymmetric Supercapacitor. J. Saudi Chem. Soc..

[76] Shah S.S., Aziz M.A., Al-Betar A.-R., Mahfoz W. (2022). Electrodeposition of Polyaniline on High Electroactive Indium Tin Oxide Nanoparticles-Modified Fluorine Doped Tin Oxide Electrode for Fabrication of High-Performance Hybrid Supercapacitor. Arab. J. Chem..

[77] Li X., Du X., Li Y., Tian X., Zheng H., Li X. (2022). Flexible and Cross-Linked N, S Co-Doped Carbon Nanofiber Nonwovens Derived from Coal Liquefaction Residue for High Performance Supercapacitors. J. Mater. Sci..

[78] Wang X., Li Y., Yang C., Cao Y., Su X., Tahir M.U. (2021). Self-Template Porous Carbon by Direct Activation of High-Ash Coal Liquefaction Residue for High-Rate Supercapacitor Electrodes. Int. J. Energy Res..

[79] Du Y., Jiang C., Song L., Gao B., Gong H., Xia W., Sheng L., Wang T., He J. (2020). Regulating Surface State of WO3 Nanosheets by Gamma Irradiation for Suppressing Hydrogen Evolution Reaction in Electrochemical N2 Fixation. Nano Res..

[80] Jiang J., Shen Q., Chen Z., Wang S. (2023). Nitrogen-Doped Porous Carbon Derived from Coal for High-Performance Dual-Carbon Lithium-Ion Capacitors. Nanomaterials.

[81] Wang X., Liu B., Wang S., Xie H., Zha Y., Huang X., Santos D.M.F., Li Y. (2023). Oxygen Self-Doped Hierarchical Porous Carbons Derived from Coal Liquefaction Residue for High-Performance Supercapacitors in Organic and Ionic Liquid-Based Electrolytes. Colloids Surf. A Physicochem. Eng. Asp..

[82] Cheng J., Lu Z., Zhao X., Chen X., Liu Y. (2021). Green Needle Coke-Derived Porous Carbon for High-Performance Symmetric Supercapacitor. J. Power Sources.

[83] Lee S.I., Mitani S., Yoon S.H., Korai Y., Mochida I. (2004). Preparation of Spherical Activated Carbon with High Electric Double-Layer Capacitance. Carbon N. Y..

[84] Bora M., Benoy S.M., Tamuly J., Saikia B.K. (2021). Ultrasonic-Assisted Chemical Synthesis of Activated Carbon from Low-Quality Subbituminous Coal and Its Preliminary Evaluation towards Supercapacitor Applications. J. Environ. Chem. Eng..

[85] Zhang Z., Liu X., Li D., Lei Y., Gao T., Wu B., Zhao J., Wang Y., Zhou G., Yao H. (2019). Mechanism of Ultrasonic Impregnation on Porosity of Activated Carbons in Non-Cavitation and Cavitation Regimes. Ultrason. Sonochem..

[86] Zhang Y., Jia J., Sun Y., Xu B., Jiang Z., Qu X., Zhang C. (2023). An Effective Strategy to Synthesize Well-Designed Activated Carbon Derived from Coal-Based Carbon Dots via Oxidation before Activation with a Low KOH Content as Supercapacitor Electrodes. Nanomaterials.

[87] Gao Z., Han X., Wang G., Liu J., Cui X., Zhang C., Wang J. (2023). Preparation of Activated Carbon Adsorption Materials Derived from Coal Gasification Fine Slag via Low-Temperature Air Activation. Gas Sci. Eng..

[88] Zhang X., Sun B., Fan X., Liang P., Zhao G., Saikia B.K., Wei X. (2022). Hierarchical Porous Carbon Derived from Coal and Biomass for High Performance Supercapacitors. Fuel.

[89] Abu Nayem S.M., Shaheen Shah S., Sultana N., Abdul Aziz M., Saleh Ahammad A.J. (2021). Electrochemical Sensing Platforms of Dihydroxybenzene: Part 2—Nanomaterials Excluding Carbon Nanotubes and Graphene. Chem. Rec..

[90] Zhong M., Wang X., Huang Y., Li L., Gao S., Tian Y., Shen W., Zhang J., Guo S. (2022). Anthracite-Derived Carbon-Based Electrode Materials for High Performance Lithium Ion Capacitors. Fuel Process. Technol..

[91] Gao Y., Zhang Y., Huang H., Deng C., Cheng Y. (2022). Low-Cost Carbon Derived from Coal-Coke for High-Performance Supercapacitors. J. Electroanal. Chem..

[92] Veluri P.S., Katchala N., Anandan S., Pramanik M., Narayansrinivasan K., Ravi B., Rao T.N. (2021). Petroleum Coke as an Efficient Single Carbon Source for High-Energy and High-Power Lithium-Ion Capacitors. Energy Fuels.

[93] Cai W., Li K., Jiang K., Lv D., Liu Y.Q., Wang D., Wang X., Lai C. (2021). Utilization of High-sulfur-Containing Petroleum Coke for Making Sulfur-Doped Porous Carbon Composite Material and Its Application in Supercapacitors. Diam. Relat. Mater..

[94] Lei J., Liu J., Tang N., Han H., Li Z., Li K., Zhai T., Chen H., Xia H. (2021). Novel Gram-Scale Synthesis of Carbon Nano-Onions from Heavy Oil for Supercapacitors. Adv. Mater. Interfaces.

[95] Mandal D., Mahapatra P.L., Kumari R., Kumbhakar P., Biswas A., Lahiri B., Chandra A., Tiwary C.S. (2021). Convert Waste Petroleum Coke to Multi-Heteroatom Self-Doped Graphene and Its Application as Supercapacitors. Emergent Mater..

[96] Hou Y., Sun Q., Du H. (2023). Fabrication of Nickel Sulfide/Hierarchical Porous Carbon from Refining Waste for High-Performance Supercapacitor. J. Alloys Compd..

[97] Pati S.K., Hwang Y., Lee H.M., Kim B.J., Park S. (2023). Porous Activated Carbon Derived from Petroleum Coke as a High-Performance Anodic Electrode Material for Supercapacitors. Carbon Lett..

[98] Zhang X., Tian X., Song Y., Wu J., Yang T., Liu Z. (2022). High-Performance Activated Carbon Cathodes from Green Cokes for Zn-Ion Hybrid Supercapacitors. Fuel.

[99] Strelkov V., Shirkunov A., Ryabov V., Chuchalina A. (2021). Granulated Activated Carbon Production Based on Petroleum Coke. IOP Conf. Ser. Earth Environ. Sci..

[100] Wu W., Zhang X., Yang J., Li J., Li X. (2020). Facile Preparation of Oxygen-Rich Activated Carbon from Petroleum Coke for Enhancing Methylene Blue Adsorption. Carbon Lett..

[101] Nanaji K., Nirogi A., Srinivas P., Anandan S., Vijay R., Bathe R.N., Pramanik M., Narayan K., Ravi B., Rao T.N. (2022). Translational Materials Research—From Laboratory to Product: A 1200 F Cylindrical Supercapacitor from Petroleum Coke Derived Activated Carbon Sheets. J. Energy Storage.

[102] Li S., Zhang J., Han J., Liu K., Wu Y. (2023). Preparation of High Specific Surface Area Activated Carbon from Semi-Coke for Carbon-Based Supercapacitor Applications. J. Phys. Conf. Ser..

[103] Li X., Zhao L., He T., Zhang M., Wang Z., Zhang B., Weng X. (2022). Highly Conductive, Hierarchical Porous Ultra-Fine Carbon Fibers Derived from Polyacrylonitrile/Polymethylmethacrylate/Needle Coke as Binder-Free Electrodes for High-Performance Supercapacitors. J. Power Sources.

[104] Zhang L., Duan Y., Gao R., Chen Z., Yue Y., An W., Du H. (2023). Upcycling of Refinery Solid Waste by Chemical Activation: Fabrication of Molybdenum Sulfide/Hierarchical Porous Carbon Composites for Highly Stable Supercapacitor. Fuel.

[105] Wan D., Cheng X., Shi Y., Chen Z., Liu Y., Han X., Liu Y., Zhang Z. (2021). Insights into Lead Removal in Water Using a Novel Carbonized Material Derived from the By-Product of Oil Refining: Action Mechanism and Performance Optimization. J. Chem. Technol. Biotechnol..

[106] Yang W., Yan L., Jiang B., Wang P., Li Z., Wang C., Bai H., Zhang C., Li Y. (2022). Crumpled Nitrogen-Doped Porous Carbon Nanosheets Derived from Petroleum Pitch for High-Performance and Flexible Electromagnetic Wave Absorption. Ind. Eng. Chem. Res..

[107] Kudinova A.A., Poltoratckaya M.E., Gabdulkhakov R.R., Litvinova T.E., Rudko V.A. (2022). Parameters Influence Establishment of the Petroleum Coke Genesis on the Structure and Properties of a Highly Porous Carbon Material Obtained by Activation of KOH. J. Porous Mater..

[108] Yang W., Wang P., Tu Z., Hou L., Yan L., Jiang B., Zhang C., Huang G., Yang F., Li Y. (2022). Heteroatoms-Doped Hierarchical Porous Carbon with Multi-Scale Structure Derived from Petroleum Asphalt for High-Performance Supercapacitors. Carbon N. Y..

[109] Albaiz A., Alsaidan M., Alzahrani A., Almoalim H., Rinaldi A., Jalilov A.S. (2023). Active Carbon-Based Electrode Materials from Petroleum Waste for Supercapacitors. C-Journal Carbon Res..

[110] Thomas R., Balachandran M. (2023). Fuel Coke Derived Nitrogen and Phosphorus Co-Doped Porous Graphene Structures for High-Performance Supercapacitors: The Trail towards a Brown-to-Green Transition. J. Energy Storage.

[111] Han L., Zhu X., Yang F., Liu Q., Jia X. (2021). Eco-Conversion of Coal into a Nonporous Graphite for High-Performance Anodes of Lithium-Ion Batteries. Powder Technol..

[112] Wang Z., Xing B., Zeng H., Huang G., Liu X., Guo H., Zhang C., Cao Y., Chen Z. (2021). Space-Confined Carbonization Strategy for Synthesis of Carbon Nanosheets from Glucose and Coal Tar Pitch for High-Performance Lithium-Ion Batteries. Appl. Surf. Sci..

[113] Zhang B., Wang L., Wang B., Zhai Y., Zeng S., Zhang M., Qian Y., Xu L. (2022). Petroleum Coke Derived Porous Carbon/NiCoP with Efficient Reviving Catalytic and Adsorptive Activity as Sulfur Host for High Performance Lithium—Sulfur Batteries. Nano Res..

[114] Wang H., Shi L., Yang Z., Liu J., Xu Y., Song M., Jiang J., Zhuang Q., Chen Y., Ju Z. (2023). Recycling of Waste Coal-Based Filter Materials into Carbon Anode Materials for Potassium and Sodium-Ion Batteries. Mater. Today Sustain..

[115] Gong X., Guo S., Ding Y., Lou B., Shi N., Wen F., Yang X., Li G., Wu B., Zhu W. (2022). Preparation of Mesocarbon Microbeads as Anode Material for Lithium-Ion Battery by Co-carbonization of FCC Decant Oil and Conductive Carbon Black. Fuel Process. Technol..

[116] Shi M., Xin Y., Chen X., Zou K., Jing W., Sun J., Chen Y., Liu Y. (2021). Coal-Derived Porous Activated Carbon with Ultrahigh Specific Surface Area and Excellent Electrochemical Performance for Supercapacitors. J. Alloys Compd..

[117] Tian X., Chen Z., Hou J., Li Z. (2024). Electrochemical Properties of Porous Carbon Derived from Coal Gasification Fine Ash via Low-Temperature Alkaline Fusion and KOH Activation. J. Energy Storage.

[118] Priyadharshini R., Saravanathamizhan R., Manimozhi V., Manokaran J., Balasubramanian N. (2020). Preparation of Activated Petroleum Coke for Supercapacitor Application. Energy Storage.

[119] Tian Q.-Q., Li X.-M., Xie L.-J., Su F.-Y., Yi Z.-L., Dong L., Chen C.-M. (2023). Insights into the Carbonization Mechanism of Bituminous Coal-Derived Carbon Materials for Lithium-Ion and Sodium-Ion Batteries. New Carbon Mater..

[120] Mohamed A.M.A., Dong S., Elhefnawey M., Dong G., Gao Y., Zhu K., Cao D. (2023). A Comparison of the Electrochemical Performance of Graphitized Coal Prepared by High-Temperature Heating and Flash Joule Heating as an Anode Material for Lithium and Potassium Ion Batteries. Chem. Phys. Lett..

[121] Jiang Y., He Z., Cui X., Liu Z., Wan J., Liu Y., Ma F. (2022). Hierarchical Porous Carbon Derived from Coal Tar Pitch by One Step Carbonization and Activation Combined with a CaO Template for Supercapacitors. New J. Chem..

[122] Mishra D., Zhou R., Hassan M.M., Hu J., Gates I., Mahinpey N., Lu Q. (2022). Bitumen and Asphaltene Derived Nanoporous Carbon and Nickel Oxide/Carbon Composites for Supercapacitor Electrodes. Sci. Rep..

[123] Ortiz-González J.D., Vásquez F.A., Bedoya-Lora F.E., Vargas O.A., Calderón J.A. (2023). Facile Transformation of Heavy Oil Residues into Carbon Composites Used for High-Performance Supercapacitors. Fuel Process. Technol..

[124] Li X., Jiang L., Zhou C., Liu J., Zeng H. (2015). Integrating Large Specific Surface Area and High Conductivity in Hydrogenated NiCo2O4 Double-Shell Hollow Spheres to Improve Supercapacitors. NPG Asia Mater..

[125] Mohamed A.M.A., Cao D., Chand K., Elhefnawey M. Catalytic Graphitization of Amorphous Coal at Low Temperature and Its Application in Lithium-Ion Batteries. Proceedings of the 2021 IEEE 4th International Conference on Nanoscience and Technology, ICNST 2021.

[126] Jiao S., Guo A., Wang F., Yu Y., Biney B.W., Liu H., Chen K., Liu D., Wang Z., Sun L. (2021). Sequential Pretreatments of an FCC Slurry Oil Sample for Preparation of Feedstocks for High-Value Solid Carbon Materials. Fuel.

[127] Wang J., Cui Y., Gu Y., Xu H., Shi Y., Ju Z., Zhuang Q. (2021). Coal-Based Modified Carbon for High Performance Sodium-Ion Battery. Solid State Ion..

[128] Wei C., Dang W., Li M., Ma X., Li M., Zhang Y. (2023). Hard-Soft Carbon Nanocomposite Prepared by Pyrolyzing Biomass and Coal Waste as Sodium-Ion Batteries Anode Material. Mater. Lett..

[129] Gao S., Liu L., Mao F., Zhang Z., Pan K., Zhou Z. (2022). Coal-Based Ultrathin N-Doped Carbon Nanosheets Synthesized by Molten-Salt Method for High-Performance Lithium-Ion Batteries. Nanotechnology.

[130] Chen H., Sun N., Wang Y., Soomro R.A., Xu B. (2023). One Stone Two Birds: Pitch Assisted Microcrystalline Regulation and Defect Engineering in Coal-Based Carbon Anodes for Sodium-Ion Batteries. Energy Storage Mater..

[131] Gong X., Lou B., Yu R., Zhang Z., Guo S., Li G., Wu B., Liu D. (2021). Carbonization of Mesocarbon Microbeads Prepared from Mesophase Pitch with Different Anisotropic Contents and Their Application in Lithium-Ion Batteries. Fuel Process. Technol..

[132] Cao B., Liu H., Xu B., Lei Y., Chen X., Song H. (2016). Mesoporous Soft Carbon as an Anode Material for Sodium Ion Batteries with Superior Rate and Cycling Performance. J. Mater. Chem. A.

[133] Liu C., Xiao N., Wang Y., Li H., Wang G., Dong Q., Bai J., Xiao J., Qiu J. (2018). Carbon Clusters Decorated Hard Carbon Nanofibers as High-Rate Anode Material for Lithium-Ion Batteries. Fuel Process. Technol..

[134] Wang L., Hu X. (2018). Recent Advances in Porous Carbon Materials for Electrochemical Energy Storage. Chem. Asian J..

[135] Cui J., Xi Y., Chen S., Li D., She X., Sun J., Han W., Yang D., Guo S. (2016). Prolifera-Green-Tide as Sustainable Source for Carbonaceous Aerogels with Hierarchical Pore to Achieve Multiple Energy Storage. Adv. Funct. Mater..

[136] Li T.-K., Ye X.-J., Zheng X.-H., Jia R., Liu C.-S. (2023). Theoretical Prediction of Janus TQ-Graphene as a Metallic Two-Dimensional Carbon Allotrope with Negative Poisson’s Ratios for High Capacity Sodium-Ion Batteries. ACS Appl. Nano Mater..

[137] Hou H., Qiu X., Wei W., Zhang Y., Ji X. (2017). Carbon Anode Materials for Advanced Sodium-Ion Batteries. Adv. Energy Mater..

[138] Li T.-K., Ye X.-J., Meng L., Liu C.-S. (2023). THFS-Carbon: A Theoretical Prediction of Metallic Carbon Allotrope with Half-Auxeticity, Planar Tetracoordinate Carbon, and Potential Application as Anode for Sodium-Ion Batteries. Phys. Chem. Chem. Phys..

[139] Shah S.S., Alfasane M.A., Bakare I.A., Aziz M.A., Yamani Z.H. (2020). Polyaniline and Heteroatoms–Enriched Carbon Derived from Pithophora Polymorpha Composite for High Performance Supercapacitor. J. Energy Storage.

[140] Sharghi H., Shiri P., Aberi M. (2018). An Overview on Recent Advances in the Synthesis of Sulfonated Organic Materials, Sulfonated Silica Materials, and Sulfonated Carbon Materials and Their Catalytic Applications in Chemical Processes. Beilstein J. Org. Chem..

[141] Dai Z., Cao Q., Liu H., Shi X., Wang X., Li H., Han Y., Li Y., Zhou J. (2019). Biomimetic Biomass-Bsed Carbon Fibers: Effect of Covalent-Bnd Connection on Performance of Derived Carbon Fibers. ACS Sustain. Chem. Eng..

[142] Wang H., Wang C., Xiong Y., Jin C., Sun Q. (2017). Simple Synthesis of N-Doped Interconnected Porous Carbon from Chinese Tofu for High-Performance Supercapacitor and Lithium-Ion Battery Applications. J. Electrochem. Soc..

[143] Prakash S., Kumar R., Gupta A., Chaudhary A., Chandaliya V.K., Dash P.S., Gurunathan P., Ramesha K., Kumari S., Dhakate S.R. (2022). A Process for Developing Spherical Graphite from Coal Tar as High Performing Carbon Anode for Li-Ion Batteries. Mater. Chem. Phys..

[144] Song W., Tang Y., Liu J., Xiao S., Zhang Y., Gao Y., Yang C., Liu L. (2023). Mild Pretreatment Synthesis of Coal-Based Phosphorus-Doped Hard Carbon with Extended Plateau Capacity as Anodes for Sodium-Ion Batteries. J. Alloys Compd..

[145] Sun Z., Hu Y., Zeng K., Li M., Zhao S., Zhang J. (2023). Turn “Waste” Into Wealth: MoO2@coal Gangue Electrocatalyst with Amorphous/Crystalline Heterostructure for Efficient Li–O2 Batteries. Small.

[146] Lithium-Ion Battery Manufacturing Capacity, 2022–2030—Charts—Data & Statistics—IEA. https://www.iea.org/data-and-statistics/charts/lithium-ion-battery-manufacturing-capacity-2022-2030.

[147] (2021). The Role of Critical Minerals in Clean Energy Transitions.

[148] Graphite Market—Westwater Resources. https://westwaterresources.net/minerals-portfolio/graphite-market/.

[149] Zhang J., Liang C., Dunn J.B. (2023). Graphite Flows in the U.S.: Insights into a Key Ingredient of Energy Transition. Environ. Sci. Technol..

[150] Li H., Zhu T., Chen X., Liu H., He G. (2022). Improving China’s Global Lithium Resource Development Capacity. Front. Environ. Sci..

[151] Wu C., Yang Y., Zhang Y., Xu H., He X., Wu X., Chou S. (2024). Hard Carbon for Sodium-Ion Batteries: Progress, Strategies and Future Perspective. Chem. Sci..

[152] Wen Y., He K., Zhu Y., Han F., Xu Y., Matsuda I., Ishii Y., Cumings J., Wang C. (2014). Expanded Graphite as Superior Anode for Sodium-Ion Batteries. Nat. Commun..

[153] Liu H., Baumann M., Dou X., Klemens J., Schneider L., Wurba A.K., Häringer M., Scharfer P., Ehrenberg H., Schabel W. (2022). Tracing the Technology Development and Trends of Hard Carbon Anode Materials—A Market and Patent Analysis. J. Energy Storage.

[154] Karabelli D., Singh S., Kiemel S., Koller J., Konarov A., Stubhan F., Miehe R., Weeber M., Bakenov Z., Birke K.P. (2020). Sodium-Based Batteries: In Search of the Best Compromise Between Sustainability and Maximization of Electric Performance. Front. Energy Res..

[155] Vaalma C., Buchholz D., Weil M., Passerini S. (2018). A Cost and Resource Analysis of Sodium-Ion Batteries. Nat. Rev. Mater..

[156] Supercapacitor Activated Carbon Market Size [2024 To 2031] Share, Trend, Growth And Industry Analysis. https://www.businessresearchinsights.com/market-reports/supercapacitor-activated-carbon-market-104798.

[157] Veeramani V., Sivakumar M., Chen S.M., Madhu R., Alamri H.R., Alothman Z.A., Hossain M.S.A., Chen C.K., Yamauchi Y., Miyamoto N. (2017). Lignocellulosic Biomass-Derived, Graphene Sheet-like Porous Activated Carbon for Electrochemical Supercapacitor and Catechin Sensing. RSC Adv..

